# Simulating Metal-Imidazole Complexes

**DOI:** 10.1021/acs.jctc.4c00581

**Published:** 2024-07-31

**Authors:** Zhen Li, Subhamoy Bhowmik, Luca Sagresti, Giuseppe Brancato, Madelyn Smith, David E. Benson, Pengfei Li, Kenneth M. Merz

**Affiliations:** † Department of Chemistry, Michigan State University, East Lansing, Michigan 48824, United States; ‡ Scuola Normale Superiore, Piazza dei Cavalieri 7, I-56126 Pisa, Italy; § CSGI, Istituto Nazionale di Fisica Nucleare (INFN) Sezione di Pisa, Largo Bruno Pontecorvo 3, 56127 Pisa, Italy; ∥ Department of Biochemistry and Molecular Biology, Michigan State University, East Lansing, Michigan 48824, United States; ⊥ Department of Chemistry & Biochemistry, Calvin University, Grand Rapids, Michigan 49546, United States; # Department of Chemistry and Biochemistry, Loyola University Chicago, Chicago, Illinois 60660, United States

## Abstract

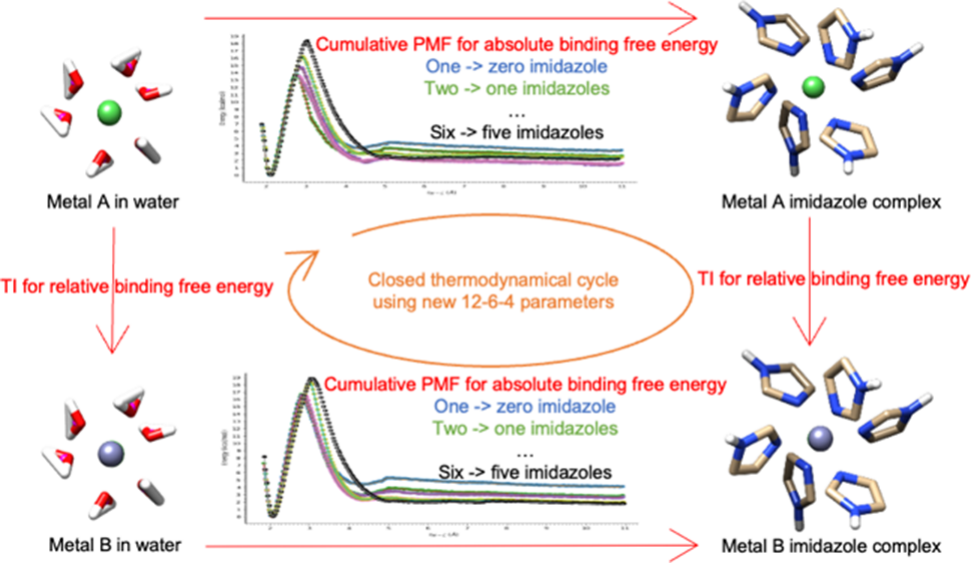

One commonly observed binding motif in metalloproteins involves the interaction between a metal ion and histidine’s imidazole side chains. Although previous imidazole-M(II) parameters established the flexibility and reliability of the 12–6–4 Lennard-Jones (LJ)-type nonbonded model by simply tuning the ligating atom’s polarizability, they have not been applied to multiple-imidazole complexes. To fill this gap, we systematically simulate multiple-imidazole complexes (ranging from one to six) for five metal ions (Co(II), Cu(II), Mn(II), Ni(II), and Zn(II)) which commonly appear in metalloproteins. Using extensive (40 ns per PMF window) sampling to assemble free energy association profiles (using OPC water and standard HID imidazole charge models from AMBER) and comparing the equilibrium distances to DFT calculations, a new set of parameters was developed to focus on energetic and geometric features of multiple-imidazole complexes. The obtained free energy profiles agree with the experimental binding free energy and DFT calculated distances. To validate our model, we show that we can close the thermodynamic cycle for metal-imidazole complexes with up to six imidazole molecules in the first solvation shell. Given the success in closing the thermodynamic cycles, we then used the same extended sampling method for six other metal ions (Ag(I), Ca(II), Cd(II), Cu(I), Fe(II), and Mg(II)) to obtain new parameters. Since these new parameters can reproduce the one-imidazole geometry and energy accurately, we hypothesize that they will reasonably predict the binding free energy of higher-level coordination numbers. Hence, we did not extend the analysis of these ions up to six imidazole complexes. Overall, the results shed light on metal–protein interactions by emphasizing the importance of ligand–ligand interaction and metal-π-stacking within metalloproteins.

## 1 Introduction

Metal ions are commonly seen in proteins, maintaining essential functions for bacteria, plants, and animals, ranging from respiratory processes to proteolysis.^1−5^ The absence of specific metal ions can cause fatal deficiencies, such as carcinogenesis, severe malnutrition, and eventually death. Over 25% of proteins contain metal ions that can function in either a structural or catalytic role^6,7^ and are targets for the design of new pharmaceutical agents.^8^ Computational chemistry has become an effective tool for examining metal ion-coordinating systems in various biological systems, like proteins, nucleic acids, carbohydrates, and lipids, to understand biological structure and function.^9−13^ Among different computational techniques, force field models and methods afford the computational speed needed to study complex biomacromolecules. However, reproducing metal ions’ structural features and thermodynamic properties in water or protein systems is still challenging for these methods under the prerequisite of maintaining a low computational cost^14^ and being physically meaningful. Apart from the accurate machine learning models,^15−18^ some commonly used physically meaningful force field models^19^ include but are not limited to bonded models, nonbonded models,^20^ Drude oscillators models,^21,22^ cationic dummy atom (CDA) and CDA_pol_ models,^23,24^ and the ReaxFF model.^25^

In the bonded model, covalent bonds exist between the metal ion and the coordinated residues, in which the bond angle, dihedral, van der Waals, and electrostatic interactions are defined by classical terms.^26^ Although the bonded model can successfully replicate experimentally determined structures, it cannot simulate the change of coordination number or ligating residues in order to model catalytic metal centers or metal ion transport.^27^

The nonbonded model, in comparison, is another widely used model for metal ions, where the metal ion is represented by a sphere using both van der Waals (vdW) and Coulombic terms to interact with its surroundings.^19^ The vdW interactions are defined by the 12–6 Lennard-Jones (LJ)^28^ or Born-Mayer potential.^29^ Therefore, the 12–6 LJ nonbonded model is used extensively for its simplicity and excellent transferability.^30−32^ However, previous work revealed that the 12–6 LJ nonbonded fails to reproduce the experimental ion-oxygen distance (IOD) and hydration free energy (HFE) of the first solvation shell simultaneously due to an underestimation of the ion–water free energy.^14,19,33−35^ The deficiency of the 12–6 model is primarily because it does not include ion-induced dipole interactions, which is an essential feature in highly polarized systems. The 12–6–4 LJ model was developed to overcome this challenge by adding the *C*_4_ term to account for the ion-induced dipole interaction. The ion-induced dipole interaction is proportional to *r*^–4^, where *r* is the distance between the two particles.^36^ We have found that the 12–6–4 model can successfully reproduce the experimental HFE and IOD simultaneously for various metal ions and in different water models.^14,19−27,30−43^

Since the 12–6–4 model is easy to apply, computationally efficient, and accurately describes the interactions between the metal ion and its coordinating ligands, it is an excellent model for simulating metal ions in molecular dynamics (MD) simulations. In earlier work,^44,45^ the 12–6–4 Lennard-Jones (LJ)-type nonbonded model has successfully simulated metal ion systems using the Potential of Mean Force (PMF) method using a modified polarizability of the metal-chelating nitrogen.^44^ This work parametrized the HID (δ-nitrogen protonated) imidazole molecules against the experimental values for 11 metals (Ag(II), Ca(II), Cd(II), Co(II), Cu(II), Cu(I), Fe(II), Mg(II), Mn(II), Ni(II), and Zn(II)) in conjunction with the commonly used OPC water model.^46^ To explore the capability of these new parameters, we selected five ions (Co(II), Cu(II), Mn(II), Ni(II), and Zn(II)) with experimental values available^47^ for a detailed analysis of multiple-imidazole interactions from the list of 11 ions. By computing these five ions’ absolute binding free energies to imidazole(s) (ranging from one to six) and comparing the results with the experiment, this parametrization routine of using PMFs with extended simulation (40 ns per window) was shown to be reliable at producing transferable parameters. These new parameters can then serve as good priors to predicting the metal-multiple-imidazole interactions that currently lack experimental data.

To explore the new parameter’s ability to accurately model the addition of multiple imidazoles to a metal center, we used thermodynamic cycles to demonstrate that we can model multiple-imidazole complexes up to six ligands. Finally, due to the lack of metal–ligand distance information, DFT calculations were conducted to obtain estimates for the metal-imidazole distances in multiple-imidazole complexes. The derived 12–6–4 models are compatible with the AMBER class of force fields and with the recommended OPC water model to allow a range of applications. We hope that the derived parameters will be useful in studying metalloprotein structure and function and for transition metal ion transport.

## 2 Computational Methods

### 2.1 Optimization of the 12–6–4 Potential

**Figure 1:**
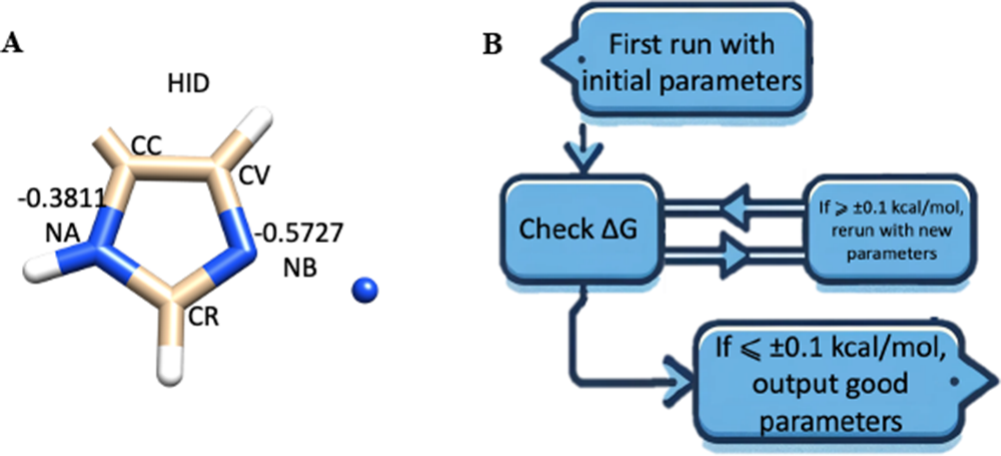
(A) Illustration and comparison of the charge distribution for both HID imidazole molecules. Heavy atom-type names in AMBER are also marked, where NA denotes the atom type of δ-nitrogen, and NB denotes the atom type of ε-nitrogen. Here, Zn(II) was used as an example, so the distance between ion and ε-nitrogen is 2.06 Å. (B) Illustration of the parametrization process.

In this work, we have used a 12–6–4 nonbonded model along with the AMBER force field:
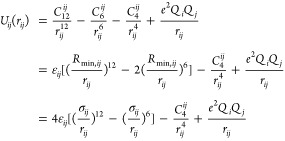
1where *e* depicts the proton charge while *Q*_*i*_ and *Q*_*j*_ represent the partial charge of atoms *i* and *j*, where *i* and *j* are the indices of the ion and one of the ligands, respectively. The Coulomb pair potential was utilized to represent the electrostatic interaction between atoms *i* and *j*. In contrast, the classic 12–6 LJ potential plus an extra *r*^4^ term represented the van der Waals interactions. The *C*_4_ terms between water and ions were taken from our previous studies.^20,36,40^ The *C*_4_ terms between histidine and metal ions were optimized based on the following equation:
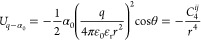
2where α_0_ is an atom type-dependent polarizability, which makes the *C*_4_ different between ligands while the metal binds to water molecules and imidazole (which mimics the side chain of histidine) simultaneously. θ represents the angle between the ion-ligand line and the induced dipole direction, which, in our case, is always zero. PMF calculations were used to optimize the pairwise parameters to reproduce the experimental free energies of each metal bound to imidazole. The HID charge used on imidazole molecules is described in Figure 1, which is the same as what ff19SB uses in AMBER20AmberTools21.^48^ The major features in the HID-charged histidine are the nitrogen charge values and the protonation locations. The connecting carbon (CC in Figure 1A) is not connected to histidine’s α-carbon but to hydrogen in the present work.^45^ The related RESP^49^ charge fitted mol2 file and frcmod file of this HID-charged imidazole molecule can be found at https://github.com/lizhen62017/MIRIAM/tree/main/TI/ff19sb_CC.frcmod and https://github.com/lizhen62017/MIRIAM/tree/main/TI/cooresp.mol2.

### 2.2 Parametrization Processes

A PMF simulation protocol was prepared according to the MD procedure described in Section 2.3. The parametrization was conducted iteratively, as Figure 1B indicates. For each iteration, a PMF with an umbrella sampling of either 4 or 40 ns for each window was performed to calculate the binding free energies. When the PMF-calculated interaction energy is within ±0.1 kcal/mol of the experimental binding free energy, the polarizability, associated *C*_4_ value, and simulated binding free energy will all be recorded. If the umbrella sampling does not give a binding free energy within the range of ±0.1 kcal/mol of the experimental value, a more precise estimate of the parameter can be obtained using linear extrapolation, assuming that the parameter and interaction energy follow a linear relationship. Then, a new round of PMF simulations is initiated with a newly assigned polarizability value to continue the iteration until an accurate binding free energy is obtained. Similarly, for the PLUMED runs, the distance restriction was instead performed by PLUMED during the umbrella sampling step of the PMF.^50^ The same 4 or 40 ns simulation time was used to construct a solid comparison between AMBER results.

**Figure 2:**
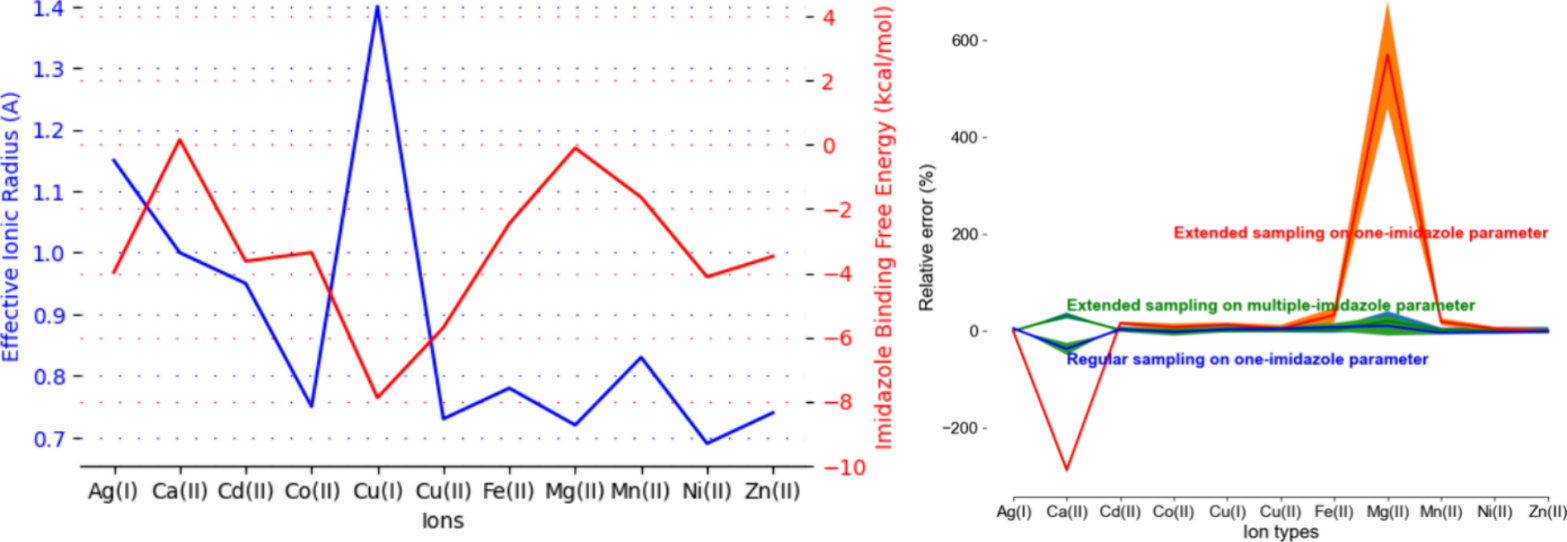
(A) Visualization of the data from Table 1. (B) Visualization of the energy data from Table 2, where extended sampling reveals the limit of one-imidazole parameters (red), which was previously not discovered by regular sampling (blue). This issue was resolved by introducing multiple-imidazole parameters (green). Color matches the color scheme for the column headers in Table 2.

### 2.3 Molecular Dynamics Simulations Using PMF Simulations

The CUDA version of PMEMD from the AMBER20^48^ package performed all the molecular dynamics (MD) simulations. The system was prepared using the tLEaP program by dissolving one ion with the designated numbers of imidazole in a 24 Å diameter octahedral box, which consists of about 5000 water molecules and corresponds to an ion concentration of approximately 10 mM. The minimization of the system was performed in three steps: (a) minimization of water molecules, with the imidazole group and ions restrained; (b) minimization of side chain hydrogens (which are imidazole hydrogens) on the nonwater molecules; (c) minimization of the whole system. Each step consists of 10,000 cycles of minimization using the steepest descent method followed by 10,000 cycles using the conjugate gradient method. In the next step, the system was heated to 300 K gradually during a 1 ns NVT simulation. A 550,000-step run was done at 300 K for system equilibration, employing the NPT ensemble. Then, a production run for 500,000 steps at 300 K under NPT conditions was performed to prepare and fully equilibrate the system. Later, a Jarzinski NMR restriction was applied to carry out a steered MD simulation between the metal ion and ε-nitrogen of one of the imidazole molecule(s) to pull them away. After the steered MD is completed, about 120 snapshots will be taken from the steered MD trajectory to start 2 ns of NPT equilibrium and a 40 ns production run with umbrella sampling as implemented in both AMBER and PLUMED. Finally, the umbrella sampling results are analyzed using the WHAM (Weighted Histogram Analysis Method) program^51^ to eventually get the PMF of one imidazole leaving the metal-imidazole complex. The process was automated in a workflow called MIRIAM (Metal-Imidazole Rational Interaction Analysis Method) and can be acquired via this link: https://github.com/lizhen62017/MIRIAM/PMF.

The Langevin thermostat with a collision frequency of 1 ps^–1^ was applied to control the temperature, and the Berendsen barostat,^52^ with a pressure relaxation time of 5 ps, was employed for the pressure control. The time step was 2 fs, and the nonbonded cutoff was 10 Å. The SHAKE^53^ algorithm was used to constrain bonds involving hydrogen atoms, and the time step was set to 2 fs. Cluster analysis was utilized to obtain the most representative structure from the MD simulations. The UCSF Chimera^34^ and VMD programs were used to visualize and prepare the figures.

### 2.4 Molecular Dynamics Simulations Using Thermodynamic Integration (TI)

To validate our PMF results, we utilized TI calculations to form a thermodynamic cycle that is supposed to close (more details below). The TI simulation starts with building a system with the two desired metal ions overlapped in the initial structure. Then the two ions were masked as “timask1” and “timask2” respectively. After this, the system will undergo the same minimization, heating, and equilibration as the MD simulation for PMF does. Then, a 12-window TI was applied to the system with up to six imidazole ligands and the metalloprotein^54^ to slowly mutate the “timask1” ion to “timask2” and compare the relative energy change. The process-related files can also be found at https://github.com/lizhen62017/MIRIAM/TI.

### 2.5 DFT Calculation on the Metal-imidazole Distances and Generation of the *C*_4_ Parameters

Because PMF studies can provide energetic and geometric information, we wanted to evaluate the new parameter’s performance in predicting metal-imidazole distances and not just binding free energies. However, since the experimental metal-imidazole distances are unknown, we used DFT calculations to estimate these quantities. Induction energies were also calculated using the Symmetry-adapted perturbation theory (SAPT) approach.^55−57^ Both DFT and SAPT are QM methods, with the latter being able to estimate the *C*_4_ parameters based on induction energies so that the ion-induced dipole interaction can be calculated.

For the 1–4 imidazole systems, the initial structures were created using the graphical interface of GaussView 6. For these systems, we kept the relative positions of the imidazole molecules similar to that of the lowest energy structure found in the PMF study. DFT calculations were carried out using Becke’s^58^ three-parameter functional and the correlation function of Lee et al. (B3LYP)^59^ and range-separated hybrid functional wB97XD.^60^ The geometry optimization of all these complexes was done using a triple-ζ basis set 6-311G++(2d,2p) with diffuse functions. Tight optimization criteria were employed with ultrafine grid integration methods. Vibrational frequency analysis was carried out to find the true minima of the complexes.

Gas phase geometries may not be able to reproduce metal ion-ligand distances in aqueous solution. So, we used the universal solvation model SMD^61^ to model an aqueous solution. From previous work, it has been seen that cluster-continuum models perform better than the continuum model for charged species, especially in calculating the hydration free energy of ions.^62−73^ Therefore, additional explicit water molecules were added to the first solvation shell to saturate the coordination of the metal ions of interest. In our calculations, we considered that these metals form high-spin, six coordinated complexes in the aqueous solution. To support our hypothesis, we calculated the energies from the optimized geometries of the metal ions that can form both high-spin and low-spin complexes (Mn(II), Ni(II), Co(II)). The high-spin systems were energetically more favorable, so only the high-spin results are presented.

The SAPT method draws a bridge between QM operators and the 12–6–4 parameters. Specifically, SAPT decomposes the molecular interaction into four different terms: electrostatic, induction, repulsion, and dispersion. Among these terms, the induction term can be interpreted as the charge-induced dipole interaction and shows a distance dependence of *1*/*r*^4^ in the long-range, which naturally fits the *C*_4_ term in the 12–6–4 model.^57^ In the present study, we calibrated the *C*_4_ parameters for metal-imidazole interactions in different complexes based on the SAPT calculated induction energy either at the equilibrium distance or along a scan of the ion-imidazole distance from 1.7 to 3.0 Å. Specifically, the induction energy can be used to derive the *C*_4_ parameters in eq 2 based on the equation *C*_4_ = *–E*_ind_*r*^4^.

## 3 Results and Discussion

### 3.1 Overview of Target Binding Free Energies, Ion Properties, and Parametrization Results

The 12–6–4 parameter set was used to reproduce the experimental metal-imidazole binding free energies compared to the experimental results. The target binding free energies computed from experimental logK values are shown in Table 1.^47^ Among all the experimental values, Mn(II), Ni(II), and Zn(II) were obtained from thermometric titration,^74−77^ Co(II) was obtained from potentiometric titration with perchloric acid,^78^ while Cu(II) was obtained from UV–vis spectra during ligand exchange of the one-ion-three-imidazole system.^79^ The experimental free energies of each ion show a high dependence on both the ionic radius and the electronic configuration. Overall, the larger effective ionic radius ions have more negative binding free energies with imidazole, with a few exceptions in the transition metal series. For these exceptions, when the d-orbitals of the ions are full or half full, they tend to have higher, meaning less negative binding free energies with imidazole, indicating a connection between d-orbital symmetry, classic Crystal-Field Stabilization Energies (CFSE), and imidazole interaction energies.^47^

**Table 1:** Experimental Interaction Energy of Ions With One Imidazole in Aqueous Environment^a^

ions	electronic configuration	effective ionic radius^80^ (Å)	imidazole binding free energy^47^ (kcal/mol)
Ag(I)	[Kr]4d^10^	1.15	–3.98
Ca(II)	[Ar]	1.00	0.16
Cd(II)	[Kr]4d^10^	0.95	–3.63
Co(II)	[Ar]3d^7^	0.75	–3.36
Cu(I)	[Ar]3d^10^	1.40	–7.89
Cu(II)	[Ar]3d^9^	0.73	–5.70
Fe(II)	[Ar]3d^6^	0.78	–2.46
Mg(II)	[Ne]	0.72	–0.10
Mn(II)	[Ar]3d^5^	0.83	–1.64
Ni(II)	[Ar]3d^7^	0.69	–4.12
Zn(II)	[Ar]3d^10^	0.74	–3.48

a Related data are visualized in Figure 2.

After investigating the correlation between ion properties and their binding free energies with imidazole molecules, the parametrization process followed the protocol summarized in the method section. As noted before, the default parameters significantly underbind to imidazole, which led us to create a set of imidazole-M(II) parameters (see Table 2).^44^ As we began to build our multiple-imidazole parameter set, we discovered that the initial protocol of 4 ns per window missed a pi-stacked intermediate (see below), which was observed only after 40 ns per window. Missing the pi-stack intermediate led to a slight overestimation of the binding affinity for one imidazole binding to an M(II) ion. However, if multiple ligands are coordinating with the metal ions, this overestimation will accumulate and lead to more significant deviations (see the one-imidazole-compatible parameter column in Table 2).^44^ Therefore, lower polarizability values for nitrogen are needed. After a reparameterization process using extended sampling, the new parameters were found to be better converged and give better predictions for multiple-imidazole systems. Although the previous parameters^44^ continue to be effective while being applied to single-imidazole systems, we recommend the newer parameter set to be used when modeling systems involve multiple imidazoles. The results of six replicate runs for each parameter set (three AMBER and three PLUMED) are summarized in Table 2.

**Table 2:**
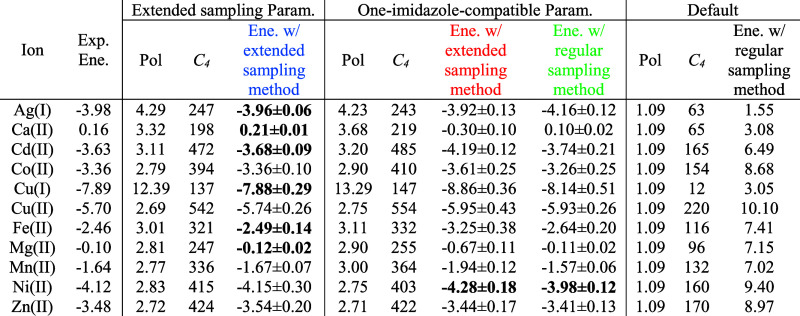
Parametrized Polarizability (Å^3^) of Nitrogen, Final *C*_4_ Value (kcal/mol·Å^4^), and Energy (kcal/mol) Comparison between the Extended Sampling Parameter, One-Imidazole-Compatible 12-6-4, and Default 12-6-4 Parameter Sets^a^^,^^b^

a **Bolded data for Ni(II)** are associated with the PMF in Figure 3, and the rest of that column has their PMFs given in Figure S1. **Bolded data on the left** are associated with PMFs given in Figure S2, and the rest of that column is associated with PMFs given in Figure S3 because these five ions were used in the thermodynamic cycle convergence analysis.

b Colors used in the column headers match the color scheme in Figure 2B.

With the exception of Ag(I), the majority of parametrized *C*_4_ values have been found to decrease slightly from the ones used in the previous one-imidazole study.^44^ After decreasing, these newly parametrized *C*_4_ values give binding free energy values closer to the experimental ones using the extended sampling protocol in both AMBER and PLUMED. By analyzing the PMF profile as illustrated in Figure 3, we found that longer simulations allow the formation of a tilted “cation-pi-stacking” conformation when the imidazole molecule is located near the second solvation shell of the metal ion.^81^ From this figure, we observe the generation of cation-pi-like interactions, while the previous runs using 4 ns did not observe this state. With the inclusion of this cation-pi-stacking state, the imidazole will have a higher energy barrier to move from the first solvation shell to the second due to the hydrogen bonds between water-imidazole. Interestingly, the imidazole-imidazole hydrogen bond does not contribute significantly to this energy shift, as the black/gray PMFs in Figure S3 indicate. This is likely due to the weak, but not negligible, deshielding effect of ε-Carbon, which contributes to a C–H NMR shift of 7.7 ppm in imidazole, where a normal C–H NMR shift should be around 7.3 ppm.^82^ In summary, this energy barrier discovered by extended sampling caused the previous parameters to overestimate the binding free energy, which led us to create the new parameter sets shown in the left column of Table 2.

**Figure 3:**
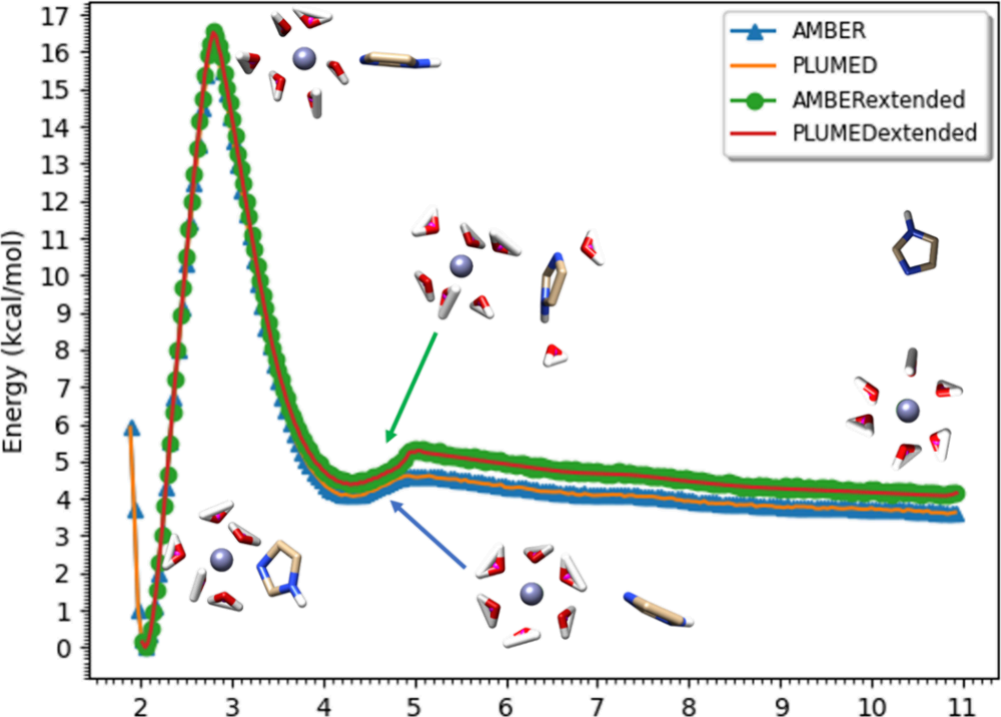
Comparison among AMBER, PLUMED, extended sampling AMBER, and extended sampling PLUMED PMF results using Ni(II) and one HID imidazole in OPC water as an example. Green arrow indicates the “cation-pi-stacking” conformation captured by the extended sampling methods for both old and new parameters. PMFs for other ions are available in Figure S1. Only one PMF of the three duplicates is presented.

### 3.2 Using the Parameters for Thermodynamic Cycle Analysis

**Figure 4:**
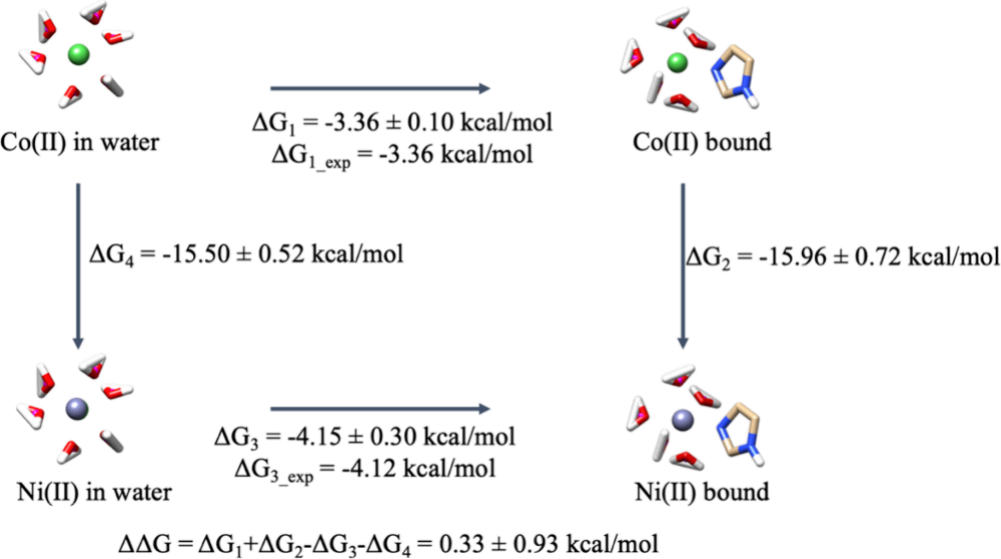
Thermodynamic cycle of mutating Co(II) to Ni(II), while the ion has one imidazole coordinating; here, Δ*G*_4_ was direct taken from a previous work.^41^

One way to further test the derived parameters is through the use of thermodynamic cycles. Using this approach provides another check on the quality of the derived parameters. In particular, we use the thermodynamic cycle shown in Figure 4 (Ni(II) → Co(II) example here) where we convert Ni(II) → Co(II) in aqueous solution and the presence of 1 to 6 imidazole molecules. Finally, the sum of the free energies around the thermodynamic cycle should be as close to 0 as possible (since free energy is a state function). With the help of this new set of parameters generated by the extended sampling method, we can better close the thermodynamic cycle. As Table 3B,C show, the previous one-imidazole-compatible and default parameters do not converge (MAE of 2.30 and 2.75 kcal/mol, which is (1.40 + 2.53 + 3.31 + 2.62 + 0.11 + 3.72)/6 and (2.05 + 3.49 + 4.45 + 4.45 + 1.76 + 0.32)/6, respectively, as the yellow column indicates), with the deviation from 0 usually being well above the uncertainty estimated from the triplicate runs. In contrast, Table 3A shows that the new parameters can generate smaller net thermodynamic cycle values (MAE of 0.61 kcal/mol, which is (0.33 + 0.18 + 0.03 + 0.37 + 1.11 + 1.64)/6 as the yellow column indicates), and the deviation from 0 is almost always within the uncertainty over the triplicate runs.

**Table 3:**
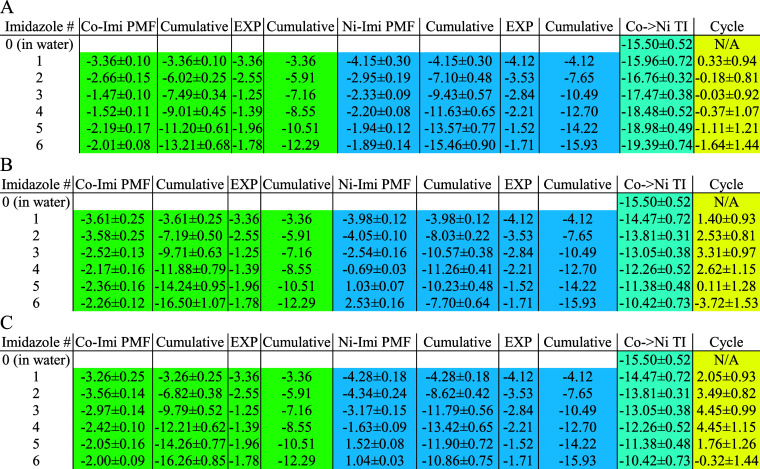
Binding Free Energy Calculated by PMF (Green and Blue, Δ*G*_1_ and Δ*G*_3_ in Figure 4)^a^^,^^b^

a From top to bottom: (A) using extended sampling PMF on extended sampling parameters, (B) using extended sampling PMF on one-imidazole-compatible parameters, and (C) using regular PMF on one-imidazole-compatible parameters.

b Relative energy calculated by TI (cyan, Δ*G*_2_ and Δ*G*_4_ in Figure 4). Thermodynamic cycle value (yellow, ΔΔ*G* in Figure 4) for the Co(II) – Ni(II) pair.

Further tests were conducted on the relative free energies between all possible 10 pairs among the 5 biologically relevant ions (Co(II), Cu(II), Mn(II), Ni(II), and Zn(II)). One example of the thermodynamic cycles using TI for the Δ*G*_2_ leg and PMF for the Δ*G*_1_ and Δ*G*_3_ legs is given in Figure 4. The associated PMFs related to Δ*G*_1_ and Δ*G*_3_ are given in Figure S3, and the remaining thermodynamic cycles are given in Figures S4∼S9. For convenience, a master table of all the data from Figures S4∼S9 is provided in Table S1.

Overall, two end points were evaluated in this study: (1) Whether the metal-imidazole(s) interaction free energy matches experimental values and (2) whether the thermodynamic cycles close. By comparing Table 3A with Table 3B, we find that the newly derived parameters greatly outperform the earlier ones using longer simulation times. Importantly, goals (1) or (2) were not as well achieved using parameters derived using 4 ns umbrella sampling, as shown in Tables 2 and 3. Overall, the extended sampling parameter sets supersede the earlier parameters and should be used in all cases. If only one imidazole is involved, the earlier parameter set can be used without significant errors, but, that said, many protein systems have multiple-imidazole side chains from His residues, making it essential to use the present parameter set.^54,83^

### 3.3 QM Calculation and Metal-Imidazole Distance Comparison

As a final validation of parameter reliability, DFT optimizations were conducted to obtain the metal-imidazole distances since no experimental distance values were available. The results are listed in Table 4. By comparing the left-side “PMF” column and the right-side “DFT” columns, the results show that the PMF-calculated distances strongly agree with the DFT calculated results with different combinations of density functional and basis set. A few exceptions still exist, such as Ag(I) and Cu(II), which always have their distances overestimated by PMF studies compared to QM, and Mn(II), which, on the other hand, has the distance values underestimated by PMF studies. For Ag(I), this is likely because the coordination number for Ag(I) is commonly overestimated in MD (see Figure S1). For Cu(II), significant Jahn–Teller axial distortions are common, making reproducibility with these spherical models difficult. The Mn(II) deviations are, however, likely due to the higher relative error from weaker binding energies rather than ligand field stabilization. For example, Hexakis(imidazole)cobalt(II) cation is high spin with a low-lying low spin excited state.^84^ Therefore, access to the Hexakis(imidazole)cobalt(II) cation low-spin excited state, which has Jahn–Teller bond length distortions,^85^ leads to variations in PMF bond lengths. The Hexakis(imidazole)manganese(II) cation has also been shown to be high-spin.,^86,87^ so with access to the low-spin state, Jahn–Teller distortions would also be possible, resulting in bond length variations for the Mn complex.

**Table 4:** Distances between the Metal Ion and ε-Nitrogen of the Imidazole^a^

ions	Imi. #	PMF/exp. distance^b^			6-311++g(2d,2p)SMD/B3LYP	6-311++g(2d,2p)SMD/WB97XD^c^
Ag(I)	1	2.34			2.13 (SDD basis set)^90^	2.13 (SDD basis set)^90^
Ca(II)	1	2.49			2.66	2.56
Cd(II)	1	2.28			2.30 (SDD basis set)^90^	2.29 (SDD basis set)^90^
Co(II)	1	2.10			2.10	2.11
	2	2.11			2.14	2.11
	3	2.13			2.13	2.11
	4	2.13			2.14	2.11
	5	2.14			2.10	2.15^c^
	6	2.14		2.15, 2.18^85^	2.20	2.16^c^
Cu(I)		1.81			1.91	1.90
Cu(II)	1	2.09			2.01	1.96
	2	2.10			2.01	2.00
	3	2.10			2.11	2.09
	4	2.10			2.09	2.07
	5	2.10			2.09	2.08^c^
	6	2.11		2.05, 2.59^91,92^	2.05	2.19^c^
Fe(II)	1	2.11		2.20, 2.20^93^	2.19	2.14
Mg(II)	1	2.11			2.17	2.16
Mn(II)	1	2.18			2.23	2.22
	2	2.19			2.29	2.26
	3	2.19			2.23	2.21
	4	2.19			2.26	2.23
	5	2.19			2.25	2.28^c^
	6	2.20		2.20, 2.27^94,95^	2.34	2.27^c^
Ni(II)	1	2.04			2.05	2.04
	2	2.04			2.07	2.05
	3	2.05			2.08	2.06
	4	2.06			2.12	2.09
	5	2.08			2.10	2.10^c^
	6	2.08		2.13^96^	2.16	2.12^c^
Zn(II)	1	2.06		1.99^d^	2.13	2.09
	2	2.08		2.01^d^	2.09	2.04
	3	2.09		2.07^d^	2.07	2.04
	4	2.09		2.13^d^	2.08	2.06
	5	2.09		2.16^d^	2.18	2.15^c^
	6	2.09	2.18, 2.19^95^	2.17^d^	2.22	2.18^c^

a If multiple-imidazole molecules are present, the average distance was taken. High spin states were used since they are energetically favored under the current level of theory and basis sets. Gaussian 16 was used.^89^

b Metal-hexaimidazole distance data are presented in “equatorial, axial” format if applicable.

c DFT optimization on MD-generated structures for faster optimization convergence.

d Optimized distances at the B3LYP-D3/6-31G* level of theory. These equilibrium distances were used to calculate the *C*_4_ values based on SAPT.

**Figure 5:**
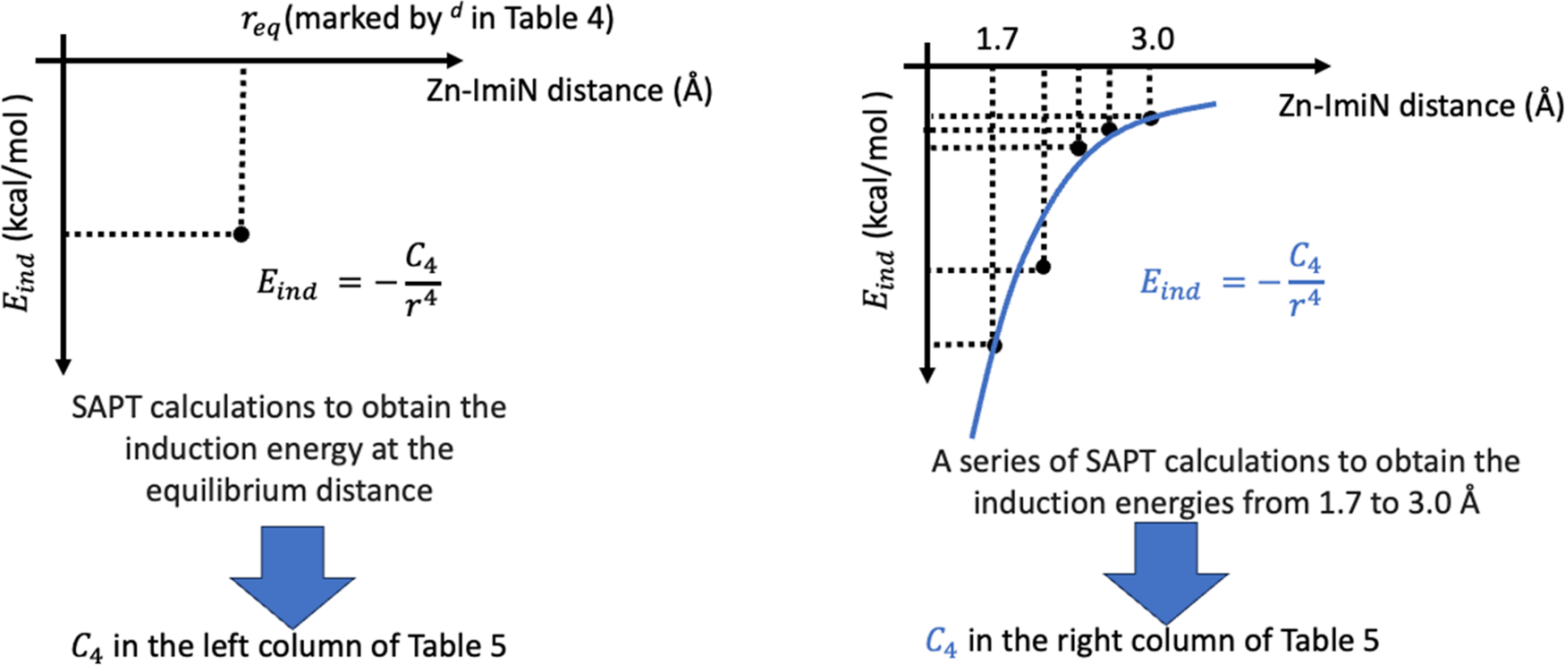
Illustration of the derivation of *C*_4_ terms based on SAPT calculations using Psi4.^88^

For Zn(II) especially, the SAPT calculation was conducted following the flowchart displayed in Figure 5, where the SAPT calculations were performed for each of the Zn(imidazole)_6−*n*_(H_2_O)_*n*_ systems, with *n* = 0∼5.

### 3.4 *C*_4_ Dependence On Ligand Number and its Correlation with Low-High Spin States

According to the induction energies calculated based on SAPT, we have found that the ion-induced dipole interaction term, which represents *C*_4_ in the 12–6–4 model, shows a strong dependency as the imidazole number increases. The result is displayed in Table 5, following the methods described in Section 2.5 and Figure 5. Comparing Tables 5 and 2, it can be concluded that the average *C*_4_ determined by SAPT is of similar magnitude to the *C*_4_ used in the simulation work (e.g., Zn(II) *C*_4_ is 424 kcal/mol·Å;^4^ the average value of the SAPT calculated *C*_4_ at the equilibrium distance is 470.8 kcal/mol·Å^4^; average *C*_4_ is 411.9 kcal/mol·Å^4^ for the fit over 1.7 to 3.0 Å). Overall, we can make two conclusions: First, the *C*_4_ value obtained using the SAPT method likely includes ion-induced dipole and also charge transfer terms. Therefore, the imidazole number will affect both the energy and metal-ligand distance significantly. Second, for the PMF parametrized *C*_4_, modeling the system with a constant value is computationally convenient, but will be less effective when facing spin state changes or Jahn–Teller effects in Cu(II). For Co(II) and Mn(II), the ground-state spin can change as the ligand field shifts from M(H_2_O)_6_^2+^ to M(imidazole)_6_^2+^. Despite the changes in ligand field stabilization energy, and enhanced imidazole affinities, the 12-6-4 model of Co(II) can well reproduce the experimental binding affinities with imidazole and agrees well with the DFT-derived bond lengths. This suggests the extended 12–6–4 model qualitatively accounts for ligand field stabilization in divalent transition metal ions. Additional work is needed to increase the precision of 12–6–4 parameters and nonspherical bond lengths derived from Jahn–Teller distortions for Cu(II) and Co(II). Ligand Field Molecular Mechanics^97−99^ is one way to further improve the 12–6–4 modeling of transition metal ions.

**Table 5:** The SAPT derived *C*_4_ parameters for the Zn(imidazole)_6−*n*_(H_2_O)_*n*_ systems, with *n* = 0∼5

systems	*C*_4_ derived based on the total induction energy at the equilibrium distance (kcal/mol·Å^4^)	*C*_4_ fitted based on the total induction energy in the range of 1.7–3.0 Å (kcal/mol·Å^4^)
Zn-Im6-Wat0	397.6	339.1
Zn-Im5-Wat1	411.9	347.5
Zn-Im4-Wat2	438.3	370.1
Zn-Im3-Wat3	490.3	428.3
Zn-Im2-Wat4	525.5	473.6
Zn-Im1-Wat5	561.4	512.9
average	470.8	411.9

## 4 Conclusions

In the present work, we have parametrized the 12–6–4 Lennard-Jones (LJ) nonbonded model for 11 biologically relevant metal ions for HID (δ-nitrogen protonated)-imidazole for the OPC water model. Among these 11 ions, five had their parameters rigorously tested using thermodynamic cycles, which demonstrated that we could both reproduce the experimental binding free energy and close the thermodynamic cycles with up to six imidazole molecules coordinating the central metal ion. Moreover, using DFT calculations, we show that the obtained *C*_4_ values reproduce M(II)-imidazole distances well. Overall, with the help of sufficient AMBER24^100^ tutorials, the obtained metal-imidazole parameter sets will provide the basis to conduct accurate MD simulations of metal-imidazole complexes at a higher level of accuracy than previously possible, both from an energy and geometric perspective. This work is also compatible with the previously established metal-acetate^101^ and metal-phosphate^102^ parameters to systematically describe ion behavior in complex protein systems. The main current limitation is the lack of parameters for ion-thiol or ion-thiolate interactions, which is largely due to the lack of experimental thermodynamic information for this class of interactions. It is worth noting that proteins that use thiol/thiolate metal interactions to bind transition metal ions make up a relatively small portion of the PDB.^103^ Importantly, the majority of ion-protein interactions are still constructed by ion-imidazole interactions, ion-acetate interactions and ion-phosphate interactions, and they present validated parameter sets that open up the possibility of accurately studying these systems.

## Supporting Information Available

PMF figures associated with Table 2 and thermodynamic cycles associated with Figure 4 and all related energies (PDF)

## References

[ref1] Andreini C.; Bertini I.; Cavallaro G.; Holliday G. L.; Thornton J. M. Metal ions in biological catalysis: from enzyme databases to general principles. Journal of Biological Inorganic Chemistry 2008, 13 (8), 1205–1218. 10.1007/s00775-008-0404-5. 18604568

[ref2] Andreini C.; Bertini I.; Cavallaro G.; Holliday G. L.; Thornton J. M. Metal-MACiE: a database of metals involved in biological catalysis. Bioinformatics 2009, 25 (16), 2088–2089. 10.1093/bioinformatics/btp256. 19369503

[ref3] Dudev T.; Lim C. Principles governing Mg, Ca, and Zn binding and selectivity in proteins. Chem. Rev. 2003, 103 (3), 773–788. 10.1021/cr020467n. 12630852

[ref4] Dudev T.; Lim C. Competition among Metal Ions for Protein Binding Sites: Determinants of Metal Ion Selectivity in Proteins. Chem. Rev. 2014, 114 (1), 538–556. 10.1021/cr4004665. 24040963

[ref5] Haas K. L.; Franz K. J. Application of Metal Coordination Chemistry To Explore and Manipulate Cell Biology. Chem. Rev. 2009, 109 (10), 4921–4960. 10.1021/cr900134a. 19715312 PMC2761982

[ref6] Klug A. The Discovery of Zinc Fingers and Their Applications in Gene Regulation and Genome Manipulation. Annu. Rev. Biochem. 2010, 79 (1), 213–231. 10.1146/annurev-biochem-010909-095056. 20192761

[ref7] Waldron K. J.; Robinson N. J. How do bacterial cells ensure that metalloproteins get the correct metal?. Nat. Rev. Microbiol 2009, 7 (1), 25–35. 10.1038/nrmicro2057. 19079350

[ref8] De Vivo M.; Masetti M.; Bottegoni G.; Cavalli A. Role of Molecular Dynamics and Related Methods in Drug Discovery. J. Med. Chem. 2016, 59 (9), 4035–4061. 10.1021/acs.jmedchem.5b01684. 26807648

[ref9] Brasen C.; Esser D.; Rauch B.; Siebers B. Carbohydrate Metabolism in Archaea: Current Insights into Unusual Enzymes and Pathways and Their Regulation. Microbiol Mol. Biol. R 2014, 78 (1), 89–175. 10.1128/MMBR.00041-13. PMC3957730 24600042

[ref10] Dacic M.; Jackman J. A.; Yorulmaz S.; Zhdanov V. P.; Kasemo B.; Cho N. J. Influence of Divalent Cations on Deformation and Rupture of Adsorbed Lipid Vesicles. Langmuir 2016, 32 (25), 6486–6495. 10.1021/acs.langmuir.6b00439. 27182843

[ref11] Maret W.; Li Y. Coordination Dynamics of Zinc in Proteins. Chem. Rev. 2009, 109 (10), 4682–4707. 10.1021/cr800556u. 19728700

[ref12] Mengistu D. H.; Bohinc K.; May S. Binding of DNA to Zwitterionic Lipid Layers Mediated by Divalent Cations. J. Phys. Chem. B 2009, 113 (36), 12277–12282. 10.1021/jp904986j. 19685861

[ref13] Sissi C.; Palumbo M. Effects of magnesium and related divalent metal ions in topoisomerase structure and function. Nucleic Acids Res. 2009, 37 (3), 702–711. 10.1093/nar/gkp024. 19188255 PMC2647314

[ref14] Li P.; Roberts B. P.; Chakravorty D. K.; Merz K. M. Jr. Rational Design of Particle Mesh Ewald Compatible Lennard-Jones Parameters for + 2 Metal Cations in Explicit Solvent. J. Chem. Theory Comput. 2013, 9 (6), 2733–2748. 10.1021/ct400146w. 23914143 PMC3728907

[ref15] Jin H.; Merz K. M. Jr. Modeling Fe(II) Complexes Using Neural Networks. J. Chem. Theory Comput. 2024, 20 (6), 2551–2558. 10.1021/acs.jctc.4c00063. 38439716 PMC10976644

[ref16] Jin H.; Merz K. M. Jr. LigandDiff: de Novo Ligand Design for 3D Transition Metal Complexes with Diffusion Models. J. Chem. Theory Comput. 2024, 20 (10), 4377–4384. 10.1021/acs.jctc.4c00232. 38743854 PMC11137811

[ref17] Jin H.; Merz K. M. Jr. Modeling Zinc Complexes Using Neural Networks. J. Chem. Inf Model 2024, 64 (8), 3140–3148. 10.1021/acs.jcim.4c00095. 38587510 PMC11040731

[ref18] Ding Y.; Huang J. DP/MM: A Hybrid Model for Zinc–Protein Interactions in Molecular Dynamics. J. Phys. Chem. Lett. 2024, 15 (2), 616–627. 10.1021/acs.jpclett.3c03158. 38198685

[ref19] Li P.; Merz K. M. Jr. Metal Ion Modeling Using Classical Mechanics. Chem. Rev. 2017, 117 (3), 1564–1686. 10.1021/acs.chemrev.6b00440. 28045509 PMC5312828

[ref20] Li P.; Song L. F.; Merz K. M. Jr. Systematic Parameterization of Monovalent Ions Employing the Nonbonded Model. J. Chem. Theory Comput. 2015, 11 (4), 1645–1657. 10.1021/ct500918t. 26574374

[ref21] Lamoureux G.; Orabi E. A. Molecular modelling of cation-pi interactions. Mol. Simulat 2012, 38 (8–9), 704–722. 10.1080/08927022.2012.696640.

[ref22] Yu H. B.; Whitfield T. W.; Harder E.; Lamoureux G.; Vorobyov I.; Anisimov V. M.; MacKerell A. D.; Roux B. Simulating Monovalent and Divalent Ions in Aqueous Solution Using a Drude Polarizable Force Field. J. Chem. Theory Comput. 2010, 6 (3), 774–786. 10.1021/ct900576a. 20300554 PMC2838399

[ref23] Aqvist J.; Warshel A. Free-Energy Relationships in Metalloenzyme-Catalyzed Reactions - Calculations of the Effects of Metal-Ion Substitutions in Staphylococcal Nuclease. J. Am. Chem. Soc. 1990, 112 (8), 2860–2868. 10.1021/ja00164a003.

[ref24] Rahnamoun A.; O’Hearn K. A.; Kaymak M. C.; Li Z.; Merz K. M. Jr.; Aktulga H. M. A Polarizable Cationic Dummy Metal Ion Model. J. Phys. Chem. Lett. 2022, 13 (23), 5334–5340. 10.1021/acs.jpclett.2c01279. 35675715

[ref25] Russo M. F.; van Duin A. C. T. Atomistic-scale simulations of chemical reactions: Bridging from quantum chemistry to engineering. Nucl. Instrum Meth B 2011, 269 (14), 1549–1554. 10.1016/j.nimb.2010.12.053.

[ref26] Case D. A.; McCammon J. A. Dynamic simulations of oxygen binding to myoglobin. Ann. N.Y. Acad. Sci. 1986, 482, 222–233. 10.1111/j.1749-6632.1986.tb20953.x. 3471106

[ref27] Case D. A.; Karplus M. Dynamics of ligand binding to heme proteins. J. Mol. Biol. 1979, 132 (3), 343–368. 10.1016/0022-2836(79)90265-1. 533895

[ref28] Hirschfelder J. O.; Ewell R. B.; Roebuck J. R. Determination of intermolecular forces from the Joule-Thomson coefficients. J. Chem. Phys. 1938, 6 (4), 205–218. 10.1063/1.1750228.

[ref29] Born M.; Mayer J. E. For the Lattice theory of Ionic crystals. Physica B 1932, 75 (1–2), 1–18. 10.1007/BF01340511.

[ref30] Aqvist J.; Warshel A. Computer simulation of the initial proton transfer step in human carbonic anhydrase I. J. Mol. Biol. 1992, 224 (1), 7–14. 10.1016/0022-2836(92)90572-2. 1312606

[ref31] Joung I. S.; Cheatham T. E. Determination of Alkali and Halide Monovalent Ion Parameters for Use in Explicitly Solvated Biomolecular Simulations. J. Phys. Chem. B 2008, 112 (30), 9020–9041. 10.1021/jp8001614. 18593145 PMC2652252

[ref32] Merz K. M. Jr Carbon dioxide binding to human carbonic anhydrase II. J. Am. Chem. Soc. 1991, 113 (2), 406–411. 10.1021/ja00002a004.

[ref33] Qiu Y.; Jiang Y.; Zhang Y.; Zhang H. Rational Design of Nonbonded Point Charge Models for Monovalent Ions with Lennard-Jones 12–6 Potential. J. Phys. Chem. B 2021, 125 (49), 13502–13518. 10.1021/acs.jpcb.1c09103. 34860517

[ref34] Zhang Y.; Jiang Y.; Peng J.; Zhang H. Rational Design of Nonbonded Point Charge Models for Divalent Metal Cations with Lennard-Jones 12–6 Potential. J. Chem. Inf Model 2021, 61 (8), 4031–4044. 10.1021/acs.jcim.1c00580. 34313132

[ref35] Zhang Y.; Jiang Y.; Qiu Y.; Zhang H. Rational Design of Nonbonded Point Charge Models for Highly Charged Metal Cations with Lennard-Jones 12–6 Potential. J. Chem. Inf Model 2021, 61 (9), 4613–4629. 10.1021/acs.jcim.1c00723. 34467756

[ref36] Li P.; Merz K. M. Jr. Taking into Account the Ion-induced Dipole Interaction in the Nonbonded Model of Ions. J. Chem. Theory Comput. 2014, 10 (1), 289–297. 10.1021/ct400751u. 24659926 PMC3960013

[ref37] Joung I. S.; Cheatham T. E. Iii Molecular dynamics simulations of the dynamic and energetic properties of alkali and halide ions using water-model-specific ion parameters. J. Phys. Chem. B 2009, 113 (40), 13279–13290. 10.1021/jp902584c. 19757835 PMC2755304

[ref38] Lamoureux G.; Roux B. Absolute Hydration Free Energy Scale for Alkali and Halide Ions Established from Simulations with a Polarizable Force Field. J. Phys. Chem. B 2006, 110 (7), 3308–3322. 10.1021/jp056043p. 16494345

[ref39] Li P.; Merz K. M. Jr. MCPB.py: A Python Based Metal Center Parameter Builder. J. Chem. Inf Model 2016, 56 (4), 599–604. 10.1021/acs.jcim.5b00674. 26913476

[ref40] Li P.; Song L. F.; Merz K. M. Jr. Parameterization of highly charged metal ions using the 12–6-4 LJ-type nonbonded model in explicit water. J. Phys. Chem. B 2015, 119 (3), 883–895. 10.1021/jp505875v. 25145273 PMC4306492

[ref41] Li Z.; Song L. F.; Li P.; Merz K. M. Systematic Parametrization of Divalent Metal Ions for the OPC3, OPC, TIP3P-FB, and TIP4P-FB Water Models. J. Chem. Theory Comput. 2020, 16, 4429 10.1021/acs.jctc.0c00194. 32510956 PMC8173364

[ref42] Li Z.; Song L. F.; Li P.; Merz K. M. Parametrization of Trivalent and Tetravalent Metal Ions for OPC3, OPC, TIP3P-FB, and TIP4P-FB Water Models. J. Chem. Theory Comput. 2021, 17, 2342 10.1021/acs.jctc.0c01320. 33793233 PMC8173366

[ref43] Sengupta A.; Li Z.; Song L. F.; Li P.; Merz K. M. Parameterization of Monovalent Ions for the OPC3, OPC, TIP3P-FB, and TIP4P-FB Water Models. J. Chem. Inf Model 2021, 61 (2), 869–880. 10.1021/acs.jcim.0c01390. 33538599 PMC8173365

[ref44] Li Z.; Song L. F.; Sharma G.; Koca Fındık B.; Merz K. M. Jr. Accurate Metal–Imidazole Interactions. J. Chem. Theory Comput. 2023, 19 (2), 619–625. 10.1021/acs.jctc.2c01081. 36584400

[ref45] Song L. F.; Sengupta A.; Merz K. M. Thermodynamics of Transition Metal Ion Binding to Proteins. J. Am. Chem. Soc. 2020, 142 (13), 6365–6374. 10.1021/jacs.0c01329. 32141296

[ref46] Izadi S.; Anandakrishnan R.; Onufriev A. V. Building Water Models: A Different Approach. J. Phys. Chem. Lett. 2014, 5 (21), 3863–3871. 10.1021/jz501780a. 25400877 PMC4226301

[ref47] Sjöberg S. Critical evaluation of stability constants of metal-imidazole and metal-histamine systems (Technical Report). Pure Appl. Chem. 1997, 69 (7), 1549–1570. 10.1351/pac199769071549.

[ref48] Case D. A.; Aktulga H. M.; Belfon K.; Ben-Shalom I.; Brozell S. R.; Cerutti D. S.; Cheatham T. E. III; Kollman P. A.; Amber 2021; University of California: San Francisco. 2021.

[ref49] Woods R. J.; Chappelle R. Restrained electrostatic potential atomic partial charges for condensed-phase simulations of carbohydrates. Journal of Molecular Structure: THEOCHEM 2000, 527 (1), 149–156. 10.1016/S0166-1280(00)00487-5. 25309012 PMC4191892

[ref50] Bonomi M.; Branduardi D.; Bussi G.; Camilloni C.; Provasi D.; Raiteri P.; Donadio D.; Marinelli F.; Pietrucci F.; Broglia R. A.; Parrinello M. PLUMED: A portable plugin for free-energy calculations with molecular dynamics. Comput. Phys. Commun. 2009, 180 (10), 1961–1972. 10.1016/j.cpc.2009.05.011.

[ref51] Grossfield A. WHAM: the weighted histogram analysis method, version 2.0. 9. Available at membrane. urmc. rochester. edu/content/wham. Accessed November 2013, 15, 2013.

[ref52] Berendsen H. J. C.; Postma J. P. M.; Gunsteren W. F. v.; DiNola A.; Haak J. R. Molecular dynamics with coupling to an external bath. J. Chem. Phys. 1984, 81 (8), 3684–3690. 10.1063/1.448118.

[ref53] Ryckaert J.-P.; Ciccotti G.; Berendsen H. J. C. Numerical integration of the cartesian equations of motion of a system with constraints: molecular dynamics of n-alkanes. J. Comput. Phys. 1977, 23 (3), 327–341. 10.1016/0021-9991(77)90098-5.

[ref54] Kakkis A.; Gagnon D.; Esselborn J.; Britt R. D.; Tezcan F. A. Metal-Templated Design of Chemically Switchable Protein Assemblies with High-Affinity Coordination Sites. Angew. Chem., Int. Ed. 2020, 59 (49), 21940–21944. 10.1002/anie.202009226. PMC7983065 32830423

[ref55] Pliego J. R. Jr; Riveros J. M. Gibbs energy of solvation of organic ions in aqueous and dimethyl sulfoxide solutions. Phys. Chem. Chem. Phys. 2002, 4 (9), 1622–1627. 10.1039/b109595a.

[ref56] Szalewicz K. Symmetry-adapted perturbation theory of intermolecular forces. WIREs Computational Molecular Science 2012, 2 (2), 254–272. 10.1002/wcms.86.

[ref57] Patkowski K. Recent developments in symmetry-adapted perturbation theory. WIREs Computational Molecular Science 2020, 10 (3), e1452 10.1002/wcms.1452.

[ref58] Becke A. D. Density-functional exchange-energy approximation with correct asymptotic behavior. Phys. Rev. A 1988, 38 (6), 3098 10.1103/PhysRevA.38.3098. 9900728

[ref59] Lee C.; Yang W.; Parr R. G. Development of the Colle-Salvetti correlation-energy formula into a functional of the electron density. Phys. Rev. B 1988, 37 (2), 785 10.1103/PhysRevB.37.785. 9944570

[ref60] Chai J.-D.; Head-Gordon M. Long-range corrected hybrid density functionals with damped atom–atom dispersion corrections. Phys. Chem. Chem. Phys. 2008, 10 (44), 6615–6620. 10.1039/b810189b. 18989472

[ref61] Marenich A. V.; Cramer C. J.; Truhlar D. G. Universal Solvation Model Based on Solute Electron Density and on a Continuum Model of the Solvent Defined by the Bulk Dielectric Constant and Atomic Surface Tensions. J. Phys. Chem. B 2009, 113 (18), 6378–6396. 10.1021/jp810292n. 19366259

[ref62] Tawa G. J.; Topol I. A.; Burt S. K.; Caldwell R. A.; Rashin A. A. Calculation of the aqueous solvation free energy of the proton. J. Chem. Phys. 1998, 109 (12), 4852–4863. 10.1063/1.477096.

[ref63] Mejıas J. A.; Lago S. Calculation of the absolute hydration enthalpy and free energy of H+ and OH–. J. Chem. Phys. 2000, 113 (17), 7306–7316. 10.1063/1.1313793.

[ref64] Rempe S. B.; Pratt L. R.; Hummer G.; Kress J. D.; Martin R. L.; Redondo A. The hydration number of Li+ in liquid water. J. Am. Chem. Soc. 2000, 122 (5), 966–967. 10.1021/ja9924750.

[ref65] Pliego J. R.; Riveros J. M. The cluster–continuum model for the calculation of the solvation free energy of ionic species. J. Phys. Chem. A 2001, 105 (30), 7241–7247. 10.1021/jp004192w.

[ref66] Zhan C.-G.; Dixon D. A. Absolute hydration free energy of the proton from first-principles electronic structure calculations. J. Phys. Chem. A 2001, 105 (51), 11534–11540. 10.1021/jp012536s.

[ref67] Zhan C.-G.; Dixon D. A. First-principles determination of the absolute hydration free energy of the hydroxide ion. J. Phys. Chem. A 2002, 106 (42), 9737–9744. 10.1021/jp014533l.

[ref68] Grabowski P.; Riccardi D.; Gomez M. A.; Asthagiri D.; Pratt L. R. Quasi-chemical theory and the standard free energy of H+ (aq). J. Phys. Chem. A 2002, 106 (40), 9145–9148. 10.1021/jp026291a.

[ref69] Zhan C.-G.; Dixon D. A. Hydration of the fluoride anion: structures and absolute hydration free energy from first-principles electronic structure calculations. J. Phys. Chem. A 2004, 108 (11), 2020–2029. 10.1021/jp0311512.

[ref70] Kelly C. P.; Cramer C. J.; Truhlar D. G. Aqueous solvation free energies of ions and ion–water clusters based on an accurate value for the absolute aqueous solvation free energy of the proton. J. Phys. Chem. B 2006, 110 (32), 16066–16081. 10.1021/jp063552y. 16898764

[ref71] Chamberlin A. C.; Cramer C. J.; Truhlar D. G. Predicting aqueous free energies of solvation as functions of temperature. J. Phys. Chem. B 2006, 110 (11), 5665–5675. 10.1021/jp057264y. 16539512

[ref72] Bryantsev V. S.; Diallo M. S.; Goddard W. A. Iii Calculation of solvation free energies of charged solutes using mixed cluster/continuum models. J. Phys. Chem. B 2008, 112 (32), 9709–9719. 10.1021/jp802665d. 18646800

[ref73] da Silva E. F.; Svendsen H. F.; Merz K. M. Explicitly Representing the Solvation Shell in Continuum Solvent Calculations. J. Phys. Chem. A 2009, 113 (22), 6404–6409. 10.1021/jp809712y. 19425558 PMC2700946

[ref74] Bauman J. E. Jr.; Wang J. C. Imidazole Complexes of Nickel(II), Copper(II), Zinc(II), and Silver(I). Inorg. Chem. 1964, 3 (3), 368–373. 10.1021/ic50013a014.

[ref75] Marsicano F.; Hancock R. D. The linear free-energy relation in the thermodynamics of complex formation. Part 2. Analysis of the formation constants of complexes of the large metal ions silver(I), mercury(II), and cadmium(II) with ligands having ‘soft’ and nitrogen-donor atoms. J. Chem. Soc., Dalton Trans. 1978, 3, 228–234. 10.1039/DT9780000228.

[ref76] Rao B.; Mathur H. B. Thermodynamics of the interaction of transition metal ions with histamine. Journal of Inorganic and Nuclear Chemistry 1971, 33 (3), 809–816. 10.1016/0022-1902(71)80481-5.

[ref77] Reddy P. R.; Rao V. B. M. Role of secondary ligands in the structure and stability of metal—cytidine complexes in solution. Polyhedron 1985, 4 (9), 1603–1609. 10.1016/S0277-5387(00)87235-6.

[ref78] Lumme P.; Virtanen P. Thermodynamics of the complexation of imidazole with divalent copper, nickel, cadmium, zinc, and cobalt ions in aqueous sodium perchlorate solutions. Acta Chem. Scand. 1974, 28a, 1055–1067. 10.3891/acta.chem.scand.28a-1055.

[ref79] Sorrell T. N.; Borovik A. S. Synthesis, structure, and spectroscopic properties of an unusual copper(I) dimer having imidazole ligands. A model for the carbonyl derivative of hemocyanin and implications for the structure of deoxyhemocyanin. J. Am. Chem. Soc. 1987, 109 (14), 4255–4260. 10.1021/ja00248a020.

[ref80] Couture A. M.; Laidler K. J. The partial molal volumes of ions in aqueous solution: I. dependence on charge and radius. Can. J. Chem. 1956, 34 (9), 1209–1216. 10.1139/v56-158.

[ref81] Turupcu A.; Tirado-Rives J.; Jorgensen W. L. Explicit Representation of Cation−π Interactions in Force Fields with 1/r4 Nonbonded Terms. J. Chem. Theory Comput. 2020, 16 (11), 7184–7194. 10.1021/acs.jctc.0c00847. 33048555 PMC7935430

[ref82] Pekmez N. Ö.; Can M.; Yildiz A. Spectroscopic and electrochemical observation of hydrogen-bonded imidazole and 2-aminoimidazole clusters. Acta Chim. Slov. 2007, 54, 131–139.

[ref83] Gilston B. A.; Skaar E. P.; Chazin W. J. Binding of transition metals to S100 proteins. Sci. China Life Sci. 2016, 59 (8), 792–801. 10.1007/s11427-016-5088-4. 27430886 PMC5123432

[ref84] Chen L.; Zhou J.; Cui H.-H.; Yuan A.-H.; Wang Z.; Zhang Y.-Q.; Ouyang Z.-W.; Song Y. Slow magnetic relaxation influenced by change of symmetry from ideal Ci to D3d in cobalt(ii)-based single-ion magnets. Dalton Transactions 2018, 47 (8), 2506–2510. 10.1039/C7DT04651K. 29384533

[ref85] Yu J.-H.; Lu J.; Xu Y.; Zhang X.; Xu J.-Q. Supramolecular structures and fluorescence properties of three transition-metal complexes. Inorg. Chim. Acta 2006, 359 (10), 3205–3211. 10.1016/j.ica.2006.02.042.

[ref86] García-Rubio I.; Angerhofer A.; Schweiger A. EPR and HYSCORE investigation of the electronic structure of the model complex Mn(imidazole)6: Exploring Mn(II)–imidazole binding using single crystals. J. Magn. Reson. 2007, 184 (1), 130–142. 10.1016/j.jmr.2006.09.013. 17055309

[ref87] Un S. Structure and Nature of Manganese(II) Imidazole Complexes in Frozen Aqueous Solutions. Inorg. Chem. 2013, 52 (7), 3803–3813. 10.1021/ic302415s. 23510244

[ref88] Smith D. G. A.; Burns L. A.; Simmonett A. C.; Parrish R. M.; Schieber M. C.; Galvelis R.; Kraus P.; Kruse H.; Di Remigio R.; Alenaizan A.; et al. PSI4 1.4: Open-source software for high-throughput quantum chemistry. J. Chem. Phys. 2020, 152 (18), 184108 10.1063/5.0006002. 32414239 PMC7228781

[ref89] Gaussian 16 Rev. C.01; Gaussian, Inc.: Wallingford, CT, 2016.

[ref90] Andrae D.; Häußermann U.; Dolg M.; Stoll H.; Preuß H. Energy-adjustedab initio pseudopotentials for the second and third row transition elements. Theoretica chimica acta 1990, 77 (2), 123–141. 10.1007/BF01114537.

[ref91] Sundberg R. J.; Martin R. B. Interactions of histidine and other imidazole derivatives with transition metal ions in chemical and biological systems. Chem. Rev. 1974, 74 (4), 471–517. 10.1021/cr60290a003.

[ref92] Server-Carrió J.; Escrivà E.; Folgado J.-V. Crystal and molecular structure, and electronic properties of hexakis(imidazole)copper(II) formate. Transition Metal Chemistry 1996, 21 (6), 541–545. 10.1007/BF00229708.

[ref93] Carver G.; Tregenna-Piggott P. L. W.; Barra A.-L.; Neels A.; Stride J. A. Spectroscopic and Structural Characterization of the [Fe(imidazole)6]2+ Cation. Inorg. Chem. 2003, 42 (18), 5771–5777. 10.1021/ic034110t. 12950228

[ref94] Niu S.-Y.; Zhang S.-S.; Li X.-M.; Wen Y.-H.; Jiao K. Hexaimidazolemanganese(II) terephthalate tetrahydrate. Acta Crystallographica Section E 2004, 60 (2), m209–m211. 10.1107/S160053680400090X.

[ref95] Garrett T. P. J.; Guss J. M.; Freeman H. C. Hexakis(imidazole)manganese(II) dichloride tetrahydrate, [Mn(C3H4N2)6]Cl2.4H2O, and hexakis(imidazole)zinc(II) dichloride tetrahydrate, [Zn(C3H4N2)6]Cl2.4H2O. Acta Crystallographica Section C 1983, 39 (8), 1027–1031. 10.1107/S0108270183007258.

[ref96] Ding Y.; Gao D. S.; Li S. D.; Li X. H.; Li C. L. Synthesis, crystal structure, and electrochemical properties of the complex [Ni(imidazole)6](DBSH)2 · 2DMF. Russian Journal of Coordination Chemistry 2009, 35 (9), 663–667. 10.1134/S1070328409090061.

[ref97] Deeth R. J. The ligand field molecular mechanics model and the stereoelectronic effects of d and s electrons. Coord. Chem. Rev. 2001, 212 (1), 11–34. 10.1016/S0010-8545(00)00354-4.

[ref98] Deeth R. J. A test of ligand field molecular mechanics as an efficient alternative to QM/MM for modelling metalloproteins: the structures of oxidised type I copper centres. Chem. Commun. 2006, 24, 2551–2553. 10.1039/b604290b. 16779474

[ref99] Foscato M.; Deeth R. J.; Jensen V. R. Integration of Ligand Field Molecular Mechanics in Tinker. J. Chem. Inf Model 2015, 55 (6), 1282–1290. 10.1021/acs.jcim.5b00098. 25970002

[ref100] Case D.A.; Aktulga H.M.; Belfon K.; Ben-Shalom I. Y.; Berryman J.T.; Brozell S.R.; Cerutti D.S.; Cheatham T.E. III; Cisneros G. A.; Cruzeiro V.W.D.; Darden T.A.; Forouzesh N.; Ghazimirsaeed M.; Ambasu G. G.; Giese T.; Gilson M.K.; Gohlke H.; Goetz A.W.; Harris J.; Huang Z.; Izadi S.; Izmailov S.A.; Kasavajhala K.; Kaymak M. C.; Kolossváry I.; Kovalenko A.; Kurtzman T.; Lee T.S.; Li P.; Li Z.; Lin C.; Liu J.; Luchko T.; Luo R.; Machado M.; Manathunga M.; Merz K.M.; Miao Y.; Mikhailovskii O.; Monard G.; Nguyen H.; Hearn K.A. O; Onufriev A.; Pan F.; Pantano S.; Rahnamoun A.; Roe D.R.; Roitberg A.; Sagui C.; Verdugo S. S.; Shajan A.; Shen J.; Simmerling C.L.; Skrynnikov N.R.; Smith J.; Swails J.; Walker R.C.; Wang J.; Wang J.; Wu X.; Wu Y.; Xiong Y.; Xue Y.; York D.M.; Zhao C.; Zhu Q.; Kollman P.A. Amber 2024; University of California: San Francisco, 2024.

[ref101] Jafari M.; Li Z.; Song L. F.; Sagresti L.; Brancato G.; Merz K. M. Jr. Thermodynamics of Metal–Acetate Interactions. J. Phys. Chem. B 2024, 128 (3), 684–697. 10.1021/acs.jpcb.3c06567. 38226860 PMC12203879

[ref102] Koca Fındık B.; Jafari M.; Song L. F.; Li Z.; Aviyente V.; Merz K. M. Jr. Binding of Phosphate Species to Ca2+ and Mg2+ in Aqueous Solution. J. Chem. Theory Comput. 2024, 20 (10), 4298–4307. 10.1021/acs.jctc.4c00218. 38718258 PMC11137831

[ref103] Putignano V.; Rosato A.; Banci L.; Andreini C. MetalPDB in 2018: a database of metal sites in biological macromolecular structures. Nucleic Acids Res. 2018, 46 (D1), D459–D464. 10.1093/nar/gkx989. 29077942 PMC5753354

[ref104] Boerner T. J.; Deems S.; Furlani T. R.; Knuth S. L.; Towns J. ACCESS: Advancing Innovation: NSF’s Advanced Cyberinfrastructure Coordination Ecosystem: Services & Support. In Practice and Experience in Advanced Research Computing; Association for Computing Machinery: Portland, OR, USA, 2023.

