# Supplementary material for: Simulating Metal-Imidazole Complexes
Source: J Chem Theory Comput. 2024 Jul 31;20(15):6706–16. doi: 10.1021/acs.jctc.4c00581 (PMC11325557; doi:10.1021/acs.jctc.4c00581)
Supplement: Supplementary file 1 — ct4c00581_si_001.pdf [file ct4c00581_si_001.pdf]

Supporting Information for

Simulating Metal-Imidazole Complexes

Zhen Li†&, Subhamoy Bhowmik†&, Luca Sagresti‡, Giuseppe Brancato‡, Madelyn Smith§, David E. Benson||, Pengfei Li§, Kenneth M. Merz, Jr.†#*

†Department of Chemistry, Michigan State University,

East Lansing, Michigan 48824, United States.

‡Scuola Normale Superiore, Piazza dei Cavalieri 7, I-56126 Pisa, Italy and

CSGI. Istituto Nazionale di Fisica Nucleare (INFN) sezione di Pisa,

Largo Bruno Pontecorvo 3, 56127 Pisa, Italy.

#Department of Biochemistry and Molecular Biology, Michigan State University,

East Lansing, Michigan 48824, United States.

||Department of Chemistry & Biochemistry, Calvin University, Grand Rapids, Michigan 49546, United States.

§Department of Chemistry and Biochemistry, Loyola University Chicago, Chicago, Illinois 60660, United States

(&Z.L. and S.B. contributed equally to this article)

**Corresponding Author:* Kenneth M. Merz

**Corresponding Author Email:* merz@chemistry.msu.edu

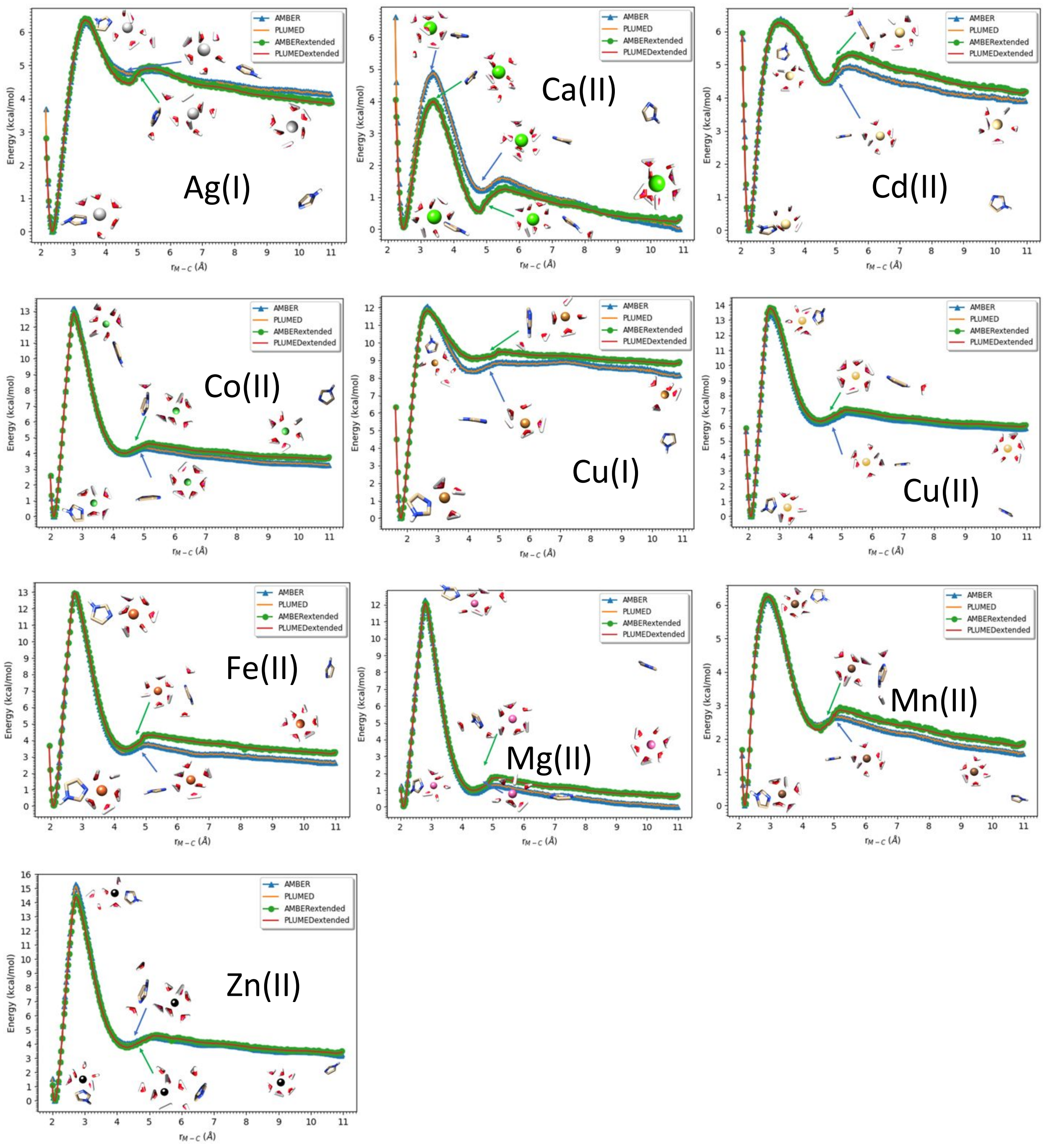

Figure S1. Comparison between AMBER, PLUMED, extended sampling AMBER, and extended sampling PLUMED PMF results for all the 10 ions other than Ni(II) which is presented in Figure 2. The green arrow indicates the "cation-pi-stacking" conformation captured by the extended sampling methods for both old and new parameters. Only one PMF of every three duplicates is presented.

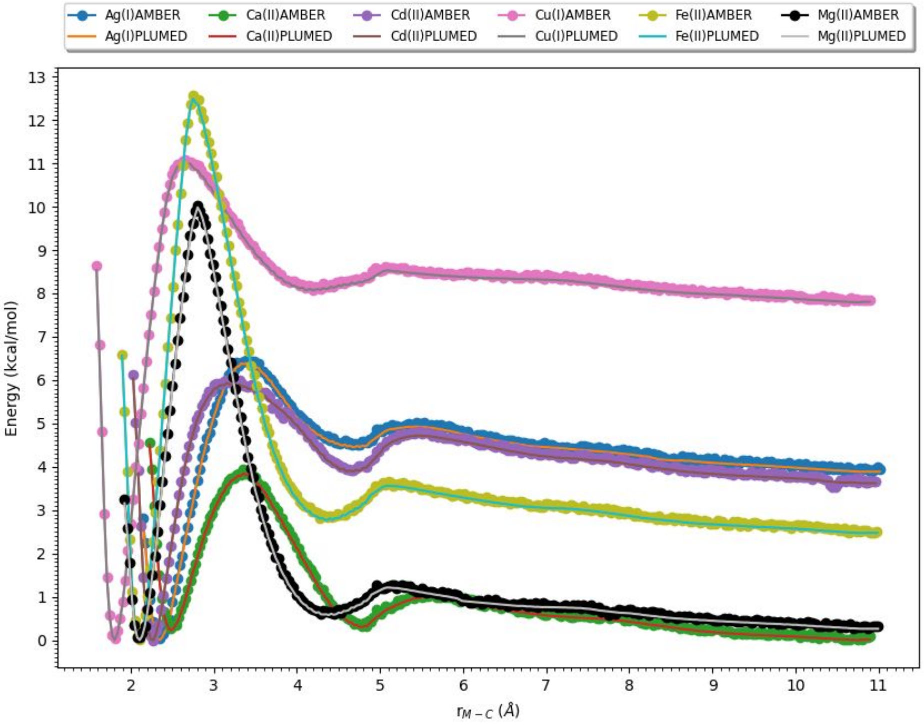

Figure S2. PMFs of the six ions with imidazoles that use the new parameter which are sampled by the extended method in both AMBER and PLUMED. Raw data is presented in the highlighted portion of Table 2. Only one PMF of every three duplicates is presented.

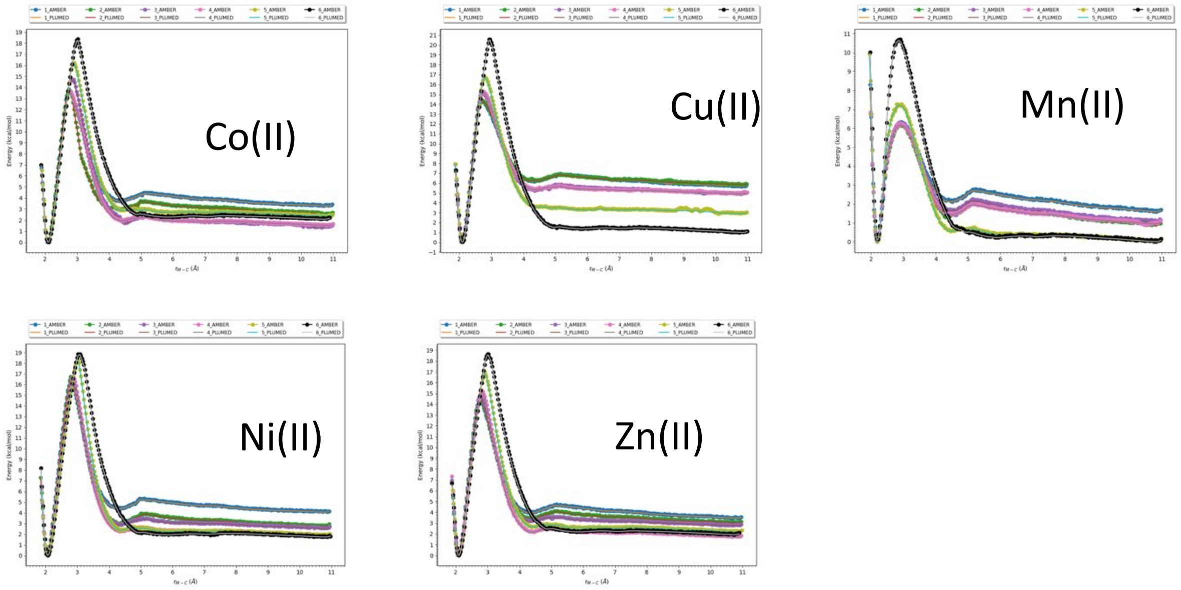

Figure S3. PMFs of the five ions with imidazoles ranging from one to six displaying the ΔG_1 and ΔG_3 in each thermodynamic cycle. Only one PMF of every three duplicates is presented.

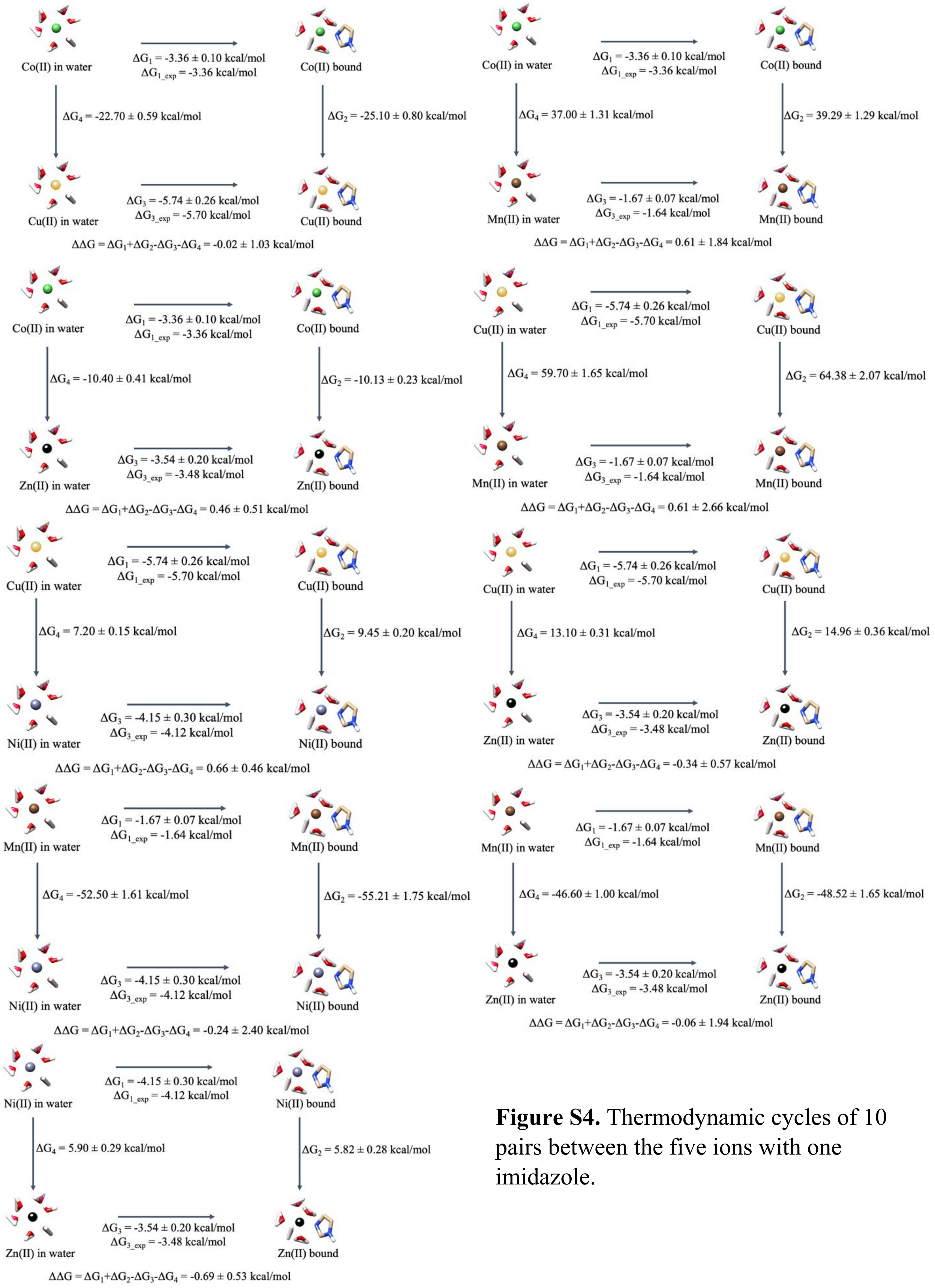

Figure S4. Thermodynamic cycles of 10 pairs between the five ions with one imidazole.

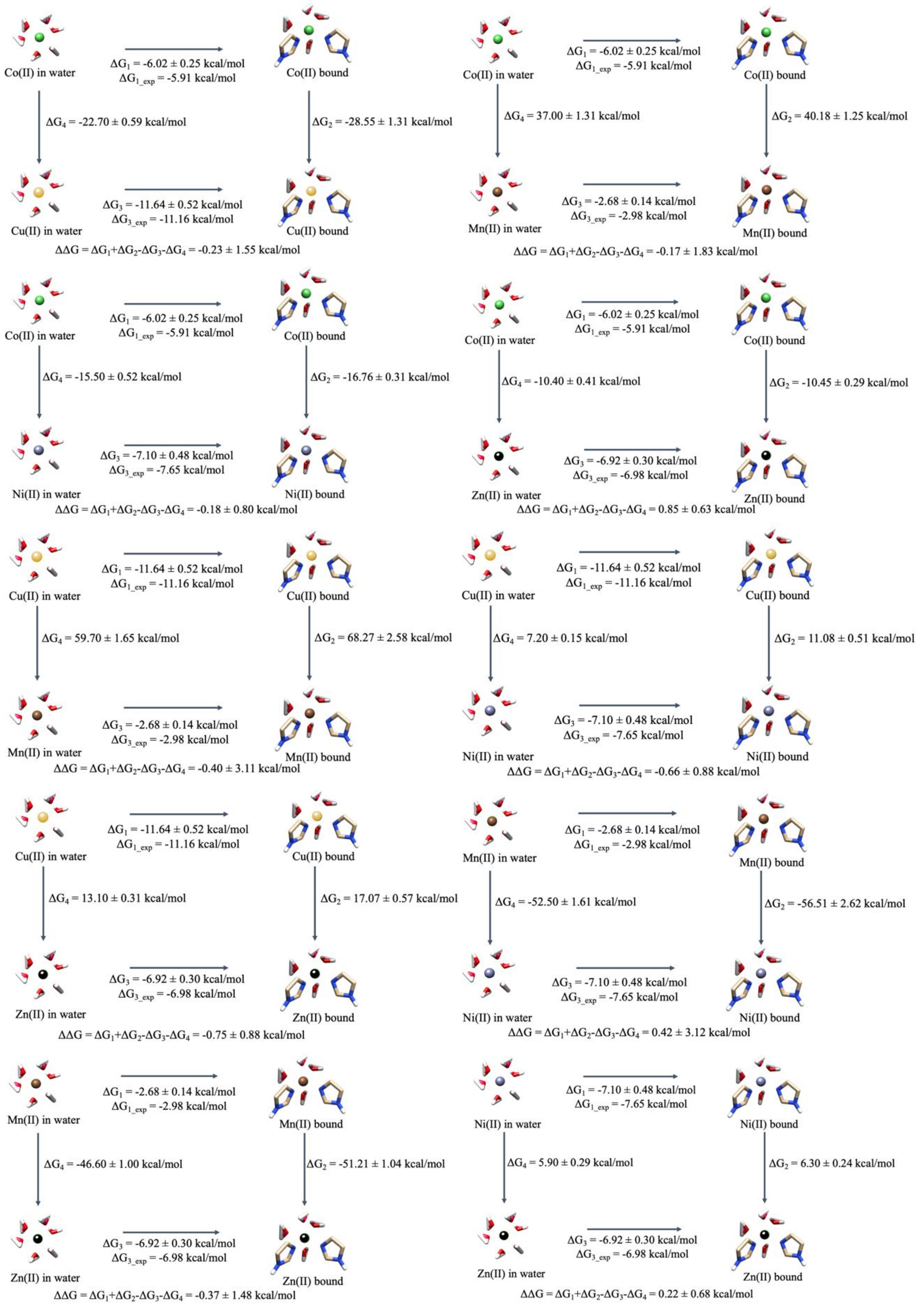

Figure S5. Thermodynamic cycles of 10 pairs between the five ions with two imidazoles.

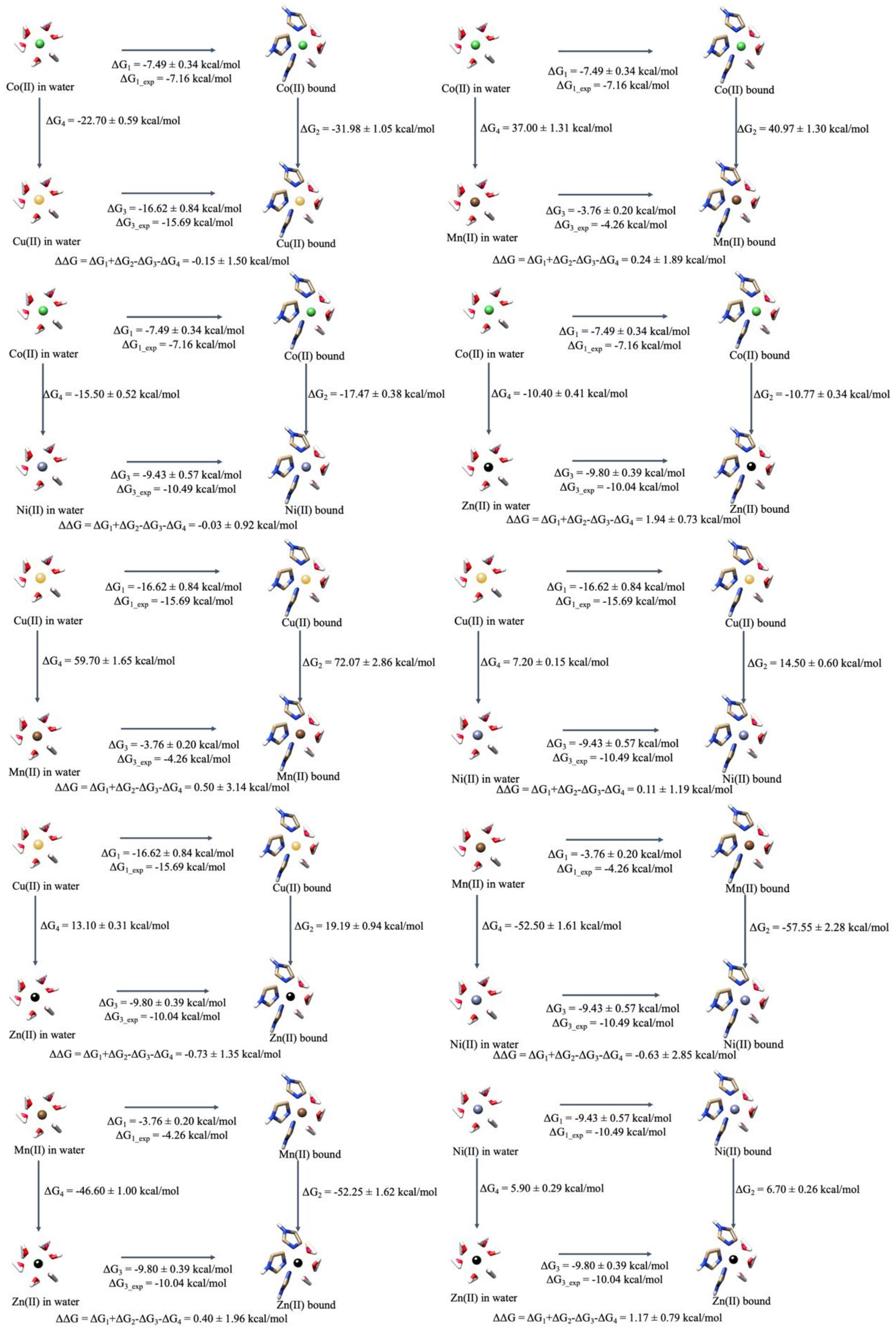

Figure S6. Thermodynamic cycles of 10 pairs between the five ions with three imidazoles.

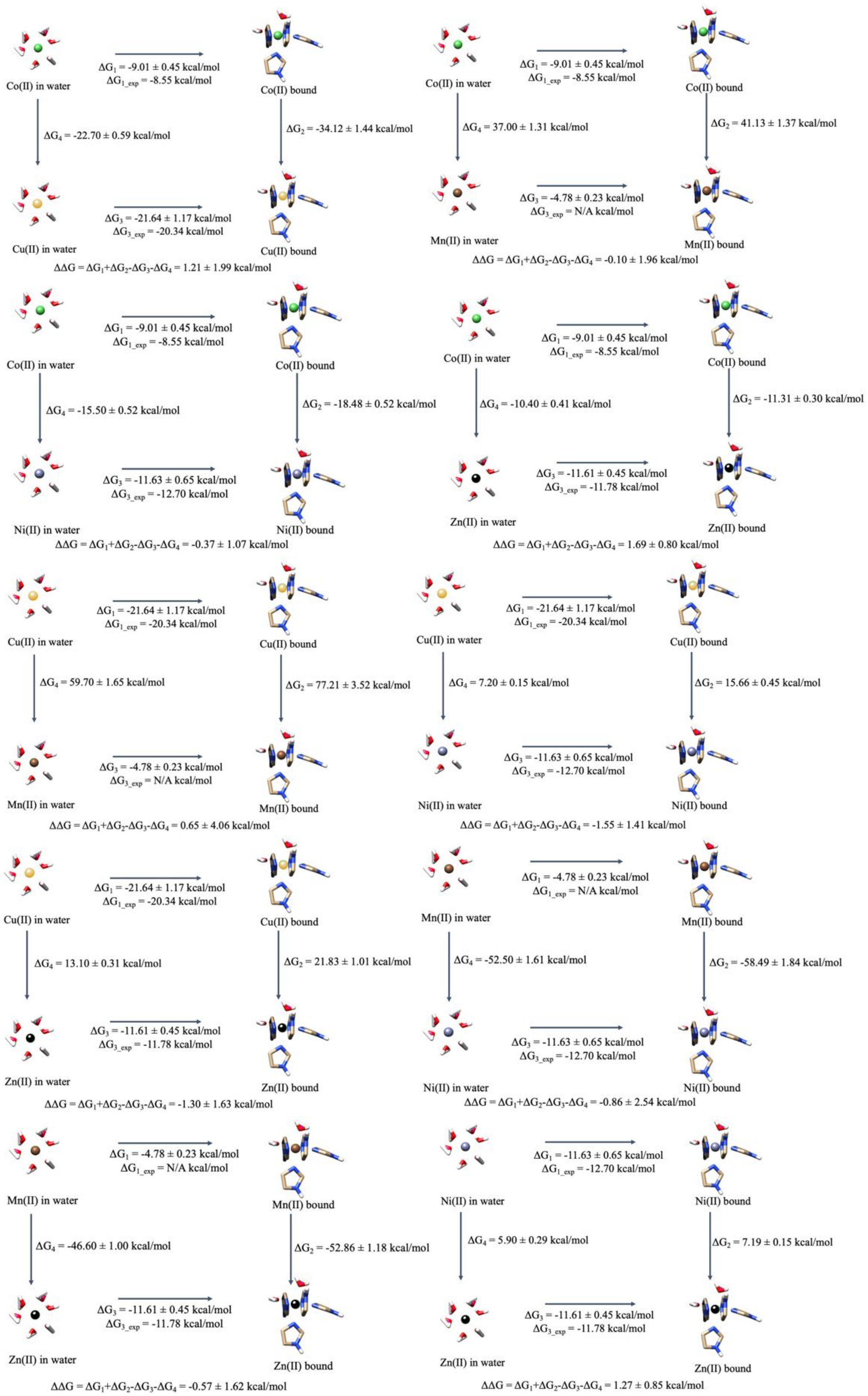

Figure S7. Thermodynamic cycles of 10 pairs between the five ions with four imidazole molecules.

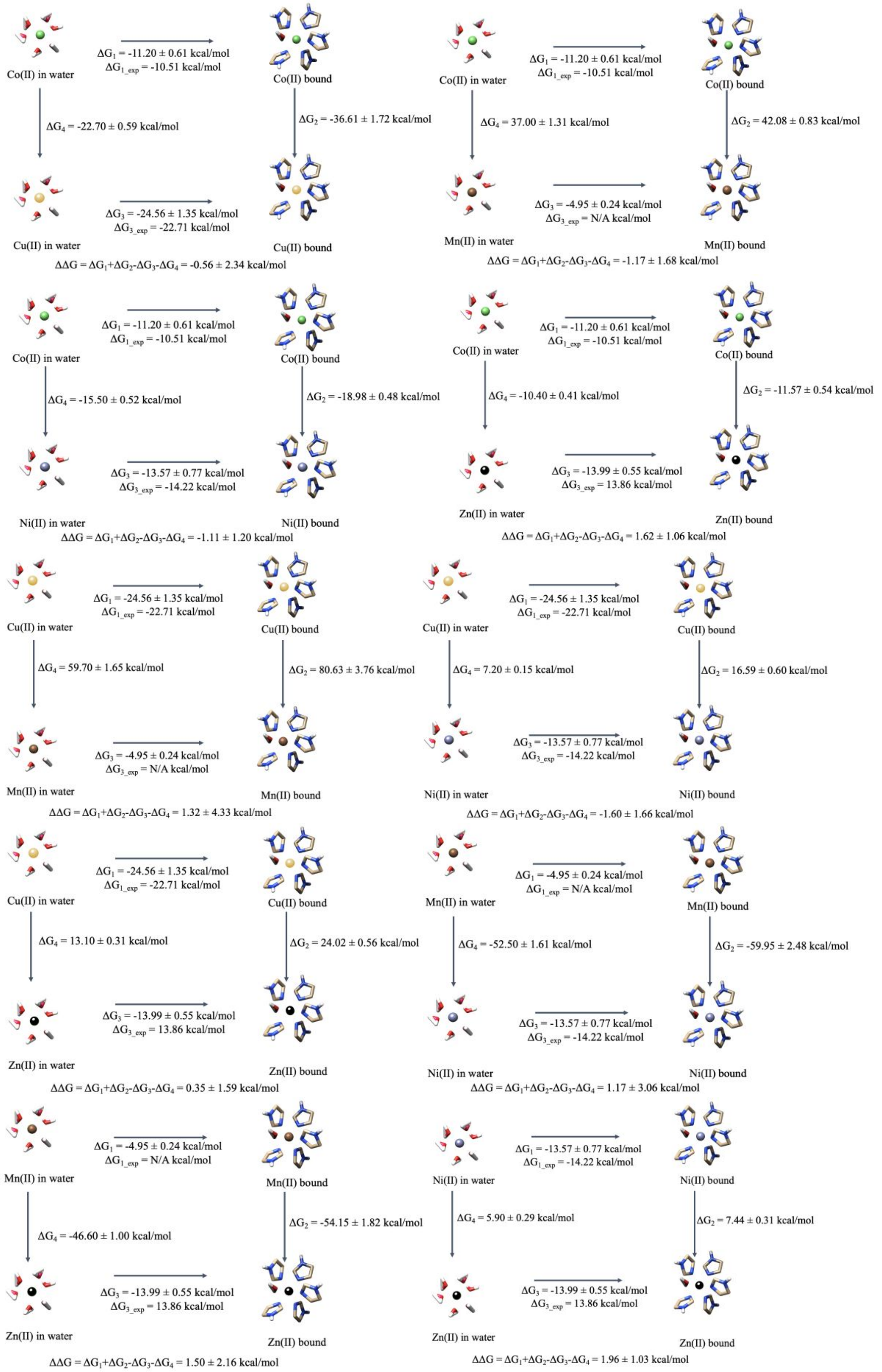

Figure S8. Thermodynamic cycles of 10 pairs between the five ions with five imidazole molecules.

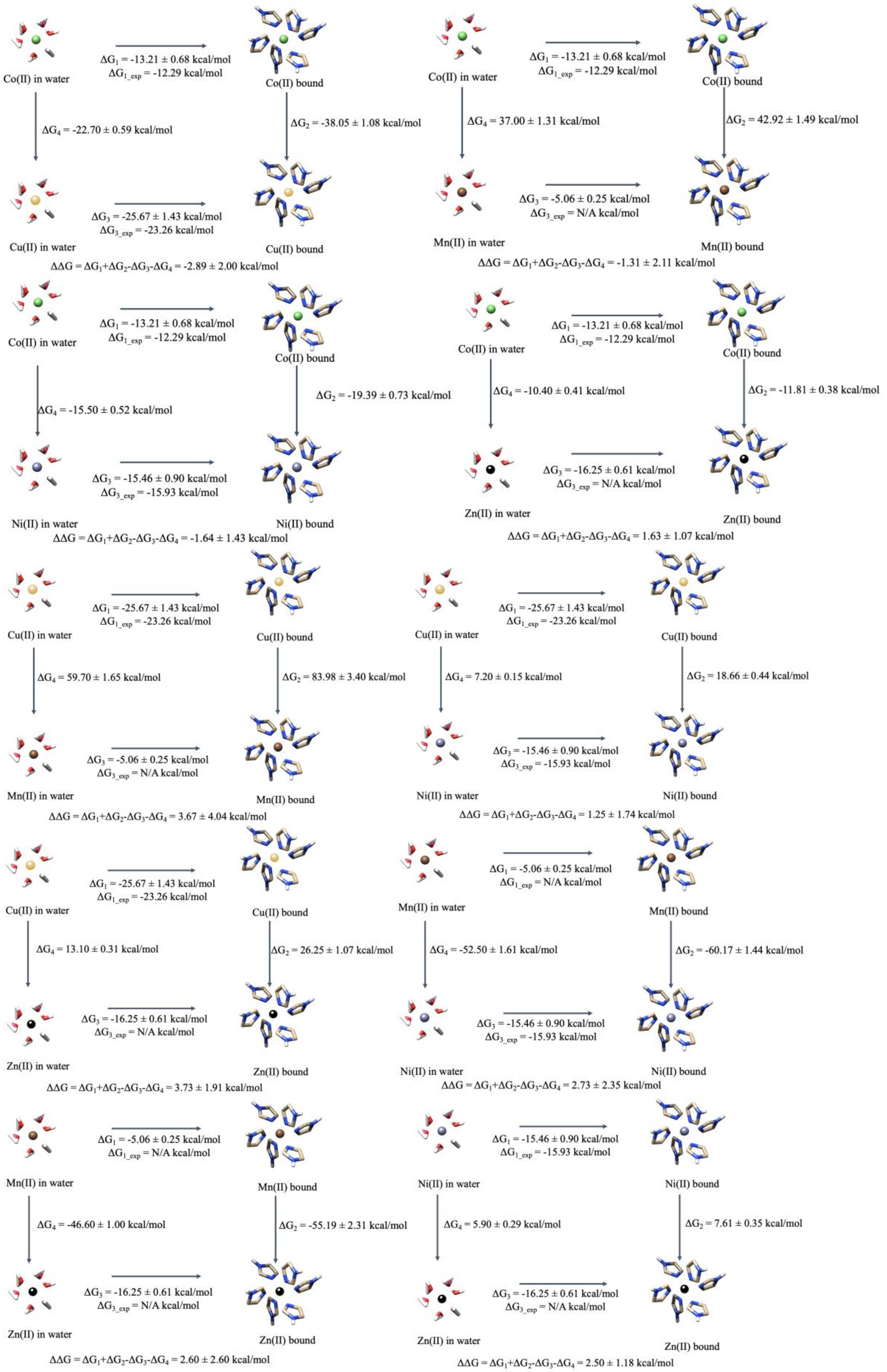

Figure S9. Thermodynamic cycles of 10 pairs between the five ions with six imidazole molecules.

Table S1. The master table containing all the ion-imidazole cluster binding free energies and the net thermodynamic cycle energies.

40ns_NEW																				
	Co	Cumulative	EXP	Cumulative	Cu	Cumulative	EXP	Cumulative	Mn	Cumulative	EXP	Cumulative	Ni	Cumulative	EXP	Cumulative	Zn	Cumulative	EXP	Cumulative
1.00	-3.36±0.10	-3.36±0.10	-3.36	-3.36	-5.74±0.26	-5.74±0.26	-5.70	-5.70	-1.67±0.07	-1.67±0.07	-1.64	-1.64	-4.15±0.30	-4.15±0.30	-4.12	-4.12	-3.54±0.20	-3.54±0.20	-3.48	-3.48
2.00	-2.66±0.15	-6.02±0.25	-2.55	-5.91	-5.90±0.26	-11.64±0.52	-5.46	-11.16	-1.01±0.07	-2.68±0.14	-1.34	-2.98	-2.95±0.19	-7.10±0.48	-3.53	-7.65	-3.38±0.11	-6.92±0.30	-3.50	-6.98
3.00	-1.47±0.10	-7.49±0.34	-1.25	-7.16	-4.98±0.33	-16.62±0.84	-4.53	-15.69	-1.08±0.06	-3.76±0.20	-1.28	-4.26	-2.33±0.09	-9.43±0.57	-2.84	-10.49	-2.88±0.10	-9.80±0.39	-3.06	-10.04
4.00	-1.52±0.11	-9.01±0.45	-1.39	-8.55	-5.02±0.33	-21.64±1.17	-4.65	-20.34	-1.02±0.04	-4.78±0.23			-2.20±0.08	-11.63±0.65	-2.21	-12.70	-1.81±0.06	-11.61±0.45	-1.74	-11.78
5.00	-2.19±0.17	-11.20±0.61	-1.96	-10.51	-2.92±0.19	-24.56±1.35	-2.37	-22.71	-0.17±0.02	-4.95±0.24			-1.94±0.12	-13.57±0.77	-1.52	-14.22	-2.38±0.11	-13.99±0.55	-2.08	-13.86
6.00	-2.01±0.08	-13.21±0.68	-1.78	-12.29	-1.11±0.08	-25.67±1.43	-0.55	-23.26	-0.11±0.01	-5.06±0.25			-1.89±0.14	-15.46±0.90	-1.71	-15.93	-2.26±0.07	-16.25±0.61		
Protein				-11.29±0.41				-16.69±0.21				-10.02±0.31				-11.20±0.32				-12.90±0.42
	CoCu	Cycle	CoMn	Cycle	CoNi	Cycle	CoZn	Cycle	CuMn	Cycle	CuNi	Cycle	CuZn	Cycle	MnNi	Cycle	MnZn	Cycle	NiZn	Cycle
0.00	-22.70±0.59		37.00±1.31		-15.50±0.52		-10.40±0.41		59.70±1.65		7.20±0.15		13.10±0.31		-52.50±1.61		-46.60±1		5.90±0.29	
1.00	-25.10±0.80	-0.02±1.03	39.29±1.30	0.61±1.85	-15.96±0.72	0.33±0.94	-10.13±0.23	0.46±0.51	64.38±2.07	0.61±2.66	9.45±0.21	0.66±0.47	14.96±0.37	-0.34±0.58	-55.21±1.76	-0.24±2.40	-48.52±1.66	-0.06±1.95	5.82±0.28	-0.69±0.53
2.00	-28.55±1.32	-0.23±1.55	40.18±1.26	-0.17±1.84	-16.76±0.32	-0.18±0.81	-10.45±0.29	0.86±0.63	68.27±2.59	-0.40±3.11	11.08±0.51	-0.66±0.88	17.07±0.58	-0.75±0.88	-56.51±2.63	0.42±3.12	-51.21±1.04	-0.37±1.48	6.30±0.24	0.22±0.68
3.00	-31.98±1.06	-0.15±1.51	40.97±1.31	0.24±1.89	-17.47±0.38	-0.03±0.92	-10.77±0.34	1.94±0.74	72.07±2.86	-0.50±3.41	14.50±0.61	0.11±1.19	19.19±0.94	-0.73±1.35	-57.55±2.28	0.63±2.86	-52.25±1.63	0.40±1.96	6.70±0.26	1.17±0.79
4.00	-34.12±1.44	1.21±1.99	41.13±1.37	-0.10±1.96	-18.48±0.52	-0.37±1.07	-11.31±0.31	1.69±0.81	77.21±3.52	0.65±4.07	15.66±0.45	-1.55±1.42	21.83±1.02	-1.30±1.64	-58.49±1.85	0.86±2.55	-52.86±1.18	0.57±1.63	7.19±0.15	1.27±0.85
5.00	-36.61±1.73	-0.56±2.35	42.08±0.84	-1.17±1.69	-18.98±0.49	-1.11±1.21	-11.57±0.54	1.62±1.06	80.63±3.77	1.32±4.33	16.59±0.60	-1.60±1.67	24.02±0.57	0.35±1.59	-59.95±2.49	1.17±3.07	-54.15±1.83	1.50±2.17	7.44±0.32	1.96±1.04
6.00	-38.05±1.09	-2.89±2.00	42.92±1.49	-1.31±2.12	-19.39±0.74	-1.64±1.44	-11.81±0.39	1.63±1.07	83.98±3.40	3.67±4.05	18.66±0.45	1.25±1.75	26.25±1.08	3.73±1.91	-60.17±1.45	2.73±2.36	-55.19±2.32	2.60±2.61	7.61±0.36	2.50±1.18
40ns_OLD																				
	Co	Cumulative	EXP	Cumulative	Cu	Cumulative	EXP	Cumulative	Mn	Cumulative	EXP	Cumulative	Ni	Cumulative	EXP	Cumulative	Zn	Cumulative	EXP	Cumulative
1.00	-3.61±0.25	-3.61±0.25	-3.36	-3.36	-5.95±0.43	-5.95±0.43	-5.70	-5.70	-1.94±0.12	-1.94±0.12	-1.64	-1.64	-3.98±0.12	-3.98±0.12	-4.12	-4.12	-3.44±0.17	-3.44±0.17	-3.48	-3.48
2.00	-3.58±0.25	-7.19±0.50	-2.55	-5.91	-5.20±0.20	-11.15±0.63	-5.46	-11.16	-1.63±0.05	-3.57±0.18	-1.34	-2.98	-4.05±0.10	-8.03±0.22	-3.53	-7.65	-3.10±0.12	-6.54±0.29	-3.50	-6.98
3.00	-2.52±0.13	-9.71±0.63	-1.25	-7.16	-4.41±0.15	-15.56±0.78	-4.53	-15.69	-1.28±0.09	-4.85±0.27	-1.28	-4.26	-2.54±0.16	-10.57±0.38	-2.84	-10.49	-2.55±0.11	-9.09±0.40	-3.06	-10.04
4.00	-2.17±0.16	-11.88±0.79	-1.39	-8.55	-5.57±0.26	-21.13±1.05	-4.65	-20.34	-0.80±0.04	-5.65±0.31			-0.69±0.03	-11.26±0.41	-2.21	-12.70	-1.58±0.11	-10.67±0.51	-1.74	-11.78
5.00	-2.36±0.16	-14.24±0.95	-1.96	-10.51	-3.88±0.15	-25.01±1.20	-2.37	-22.71	-0.09±0.01	-5.74±0.32			1.03±0.07	-10.23±0.48	-1.52	-14.22	-1.23±0.04	-11.90±0.55	-2.08	-13.86
6.00	-2.26±0.12	-16.50±1.07	-1.78	-12.29	-1.98±0.13	-26.99±1.33	-0.55	-23.26	0.19±0.01	-5.55±0.33			2.53±0.16	-7.70±0.64	-1.71	-15.93	-0.47±0.02	-12.37±0.57		
	CoCu	Cycle	CoMn	Cycle	CoNi	Cycle	CoZn	Cycle	CuMn	Cycle	CuNi	Cycle	CuZn	Cycle	MnNi	Cycle	MnZn	Cycle	NiZn	Cycle
0.00	-22.70±0.59		37.00±1.31		-15.50±0.52		-10.40±0.41		59.70±1.65		7.20±0.15		13.10±0.31		-52.50±1.61		-46.60±1		5.90±0.29	
1.00	-24.90±0.80	0.14±1.11	38.87±1.29	0.20±1.86	-14.47±0.72	1.40±0.93	-9.20±0.23	1.03±0.55	63.78±2.07	0.07±2.68	10.45±0.20	1.28±0.51	15.69±0.36	0.08±0.66	-53.34±1.75	1.20±2.39	-48.09±1.65	0.01±1.94	5.23±0.28	-1.21±0.45
2.00	-27.14±1.31	-0.48±1.65	39.97±1.25	-0.65±1.89	-13.81±0.31	2.53±0.81	-8.60±0.29	1.15±0.76	67.03±2.58	-0.25±3.13	13.38±0.51	3.06±0.85	18.53±0.57	0.82±0.95	-53.75±2.62	3.21±3.09	-48.55±1.04	1.02±1.48	5.21±0.24	-2.18±0.52
3.00	-29.37±1.05	-0.82±1.57	40.85±1.30	-1.01±1.97	-13.05±0.38	3.31±0.97	-7.99±0.34	1.79±0.91	70.27±2.86	-0.14±3.4	16.31±0.60	4.12±1.07	21.37±0.94	1.80±1.32	-53.91±2.28	4.31±2.83	-48.85±1.62	1.99±1.97	5.07±0.26	-2.31±0.67
4.00	-31.58±1.44	0.37±2.03	41.61±1.37	-1.62±2.07	-12.26±0.52	2.62±1.15	-7.38±0.30	1.81±1.07	73.18±3.52	-2.00±4.04	19.32±0.45	2.25±1.22	24.20±1.01	0.64±1.57	-53.87±1.84	4.24±2.50	-49.01±1.18	2.61±1.65	4.89±0.15	-1.60±0.73
5.00	-33.80±1.72	-0.33±2.38	42.15±0.83	-3.35±1.85	-11.38±0.48	0.11±1.28	-6.75±0.54	1.31±1.29	75.95±3.76	-3.02±4.29	22.40±0.60	0.42±1.43	27.03±0.56	0.82±1.47	-53.53±2.48	3.46±3.01	-48.98±1.82	3.78±2.17	4.63±0.31	0.40±0.85
6.00	-36.00±1.08	-2.81±2.11	42.50±1.49	-5.45±2.28	-10.42±0.73	-3.72±1.53	-6.12±0.38	0.15±1.34	78.50±3.40	-2.64±4.02	25.60±0.44	-0.89±1.55	29.89±1.07	2.17±1.83	-52.93±1.44	1.72±2.28	-48.62±2.31	4.80±2.60	4.30±0.35	3.07±0.97
4ns_OLD																				
	Co	Cumulative	EXP	Cumulative	Cu	Cumulative	EXP	Cumulative	Mn	Cumulative	EXP	Cumulative	Ni	Cumulative	EXP	Cumulative	Zn	Cumulative	EXP	Cumulative
1.00	-3.26±0.25	-3.26±0.25	-3.36	-3.36	-5.93±0.26	-5.93±0.26	-5.70	-5.70	-1.57±0.06	-1.57±0.06	-1.64	-1.64	-4.28±0.18	-4.28±0.18	-4.12	-4.12	-3.41±0.13	-3.41±0.13	-3.48	-3.48
2.00	-3.56±0.14	-6.82±0.38	-2.55	-5.91	-5.89±0.29	-11.82±0.55	-5.46	-11.16	-1.61±0.08	-3.18±0.14	-1.34	-2.98	-4.34±0.24	-8.62±0.42	-3.53	-7.65	-3.42±0.23	-6.83±0.36	-3.50	-6.98
3.00	-2.97±0.14	-9.79±0.52	-1.25	-7.16	-5.38±0.24	-17.2±0.78	-4.53	-15.69	-1.49±0.08	-4.67±0.21	-1.28	-4.26	-3.17±0.15	-11.79±0.56	-2.84	-10.49	-3.15±0.14	-9.98±0.49	-3.06	-10.04
4.00	-2.42±0.10	-12.21±0.62	-1.39	-8.55	-5.12±0.24	-22.32±1.02	-4.65	-20.34	-1.12±0.04	-5.79±0.25			-1.63±0.09	-13.42±0.65	-2.21	-12.70	-1.91±0.10	-11.89±0.58	-1.74	-11.78
5.00	-2.05±0.16	-14.26±0.77	-1.96	-10.51	-4.85±0.35	-27.17±1.36	-2.37	-22.71	-0.46±0.03	-6.25±0.27			1.52±0.08	-11.90±0.72	-1.52	-14.22	-2.39±0.10	-14.28±0.67	-2.08	-13.86
6.00	-2.00±0.09	-16.26±0.85	-1.78	-12.29	-3.91±0.26	-31.08±1.62	-0.55	-23.26	-0.25±0.02	-6.50±0.29			1.04±0.03	-10.86±0.75	-1.71	-15.93	-1.76±0.08	-16.04±0.75		
	CoCu	Cycle	CoMn	Cycle	CoNi	Cycle	CoZn	Cycle	CuMn	Cycle	CuNi	Cycle	CuZn	Cycle	MnNi	Cycle	MnZn	Cycle	NiZn	Cycle
0.00	-22.70±0.59		37.00±1.31		-15.50±0.52		-10.40±0.41		59.70±1.65		7.20±0.15		13.10±0.31		-52.50±1.61		-46.60±1		5.90±0.29	
1.00	-24.90±0.80	0.47±1.05	38.87±1.29	0.18±2.30	-14.47±0.72	2.05±0.93	-9.20±0.23	1.35±0.58	63.78±2.07	-0.28±2.66	10.45±0.20	1.60±0.40	15.69±0.36	0.07±0.55	-53.34±1.75	1.87±2.39	-48.09±1.65	0.35±1.94	5.23±0.28	-1.54±0.45
2.00	-27.14±1.31	0.56±1.58	39.97±1.25	-0.67±2.19	-13.81±0.31	3.49±0.82	-8.60±0.29	1.81±0.73	67.03±2.58	-1.31±3.11	13.38±0.51	2.98±0.86	18.53±0.57	0.44±0.92	-53.75±2.62	4.19±3.11	-48.55±1.04	1.70±1.49	5.21±0.24	-2.48±0.66
3.00	-29.37±1.05	0.74±1.52	40.85±1.30	-1.27±2.43	-13.05±0.38	4.45±0.99	-7.99±0.34	2.60±0.86	70.27±2.86	-1.96±3.39	16.31±0.60	3.70±1.14	21.37±0.94	1.05±1.34	-53.91±2.28	5.71±2.85	-48.85±1.62	3.06±1.98	5.07±0.26	-2.64±0.83
4.00	-31.58±1.44	1.23±1.95	41.61±1.37	-1.81±1.91	-12.26±0.52	4.45±1.15	-7.38±0.30	2.70±0.97	73.18±3.52	-3.05±4.02	19.32±0.45	3.22±1.29	24.20±1.01	0.67±1.57	-53.87±1.84	6.26±2.54	-49.01±1.18	3.69±1.66	4.89±0.15	-2.54±0.92
5.00	-33.80±1.72	1.81±2.39	42.15±0.83	-2.86±2.23	-11.38±0.48	1.76±1.26	-6.75±0.54	3.67±1.12	75.95±3.76	-4.67±4.33	22.40±0.60	-0.07±1.65	27.03±0.56	1.04±1.64	-53.53±2.48	4.62±3.06	-48.98±1.82	5.65±2.20	4.63±0.31	1.11±1.07
6.00	-36.00±1.08	1.52±2.20	42.50±1.49	-4.26±2.38	-10.42±0.73	-0.32±1.44	-6.12±0.38	4.06±1.22	78.50±3.40	-5.78±4.11	25.60±0.44	-1.82±1.84	29.89±1.07	1.75±2.09	-52.93±1.44	3.93±2.31	-48.62±2.31	7.52±2.64	4.30±0.35	3.58±1.15
